# Constructing and validating of m6a-related genes prognostic signature for stomach adenocarcinoma and immune infiltration: Potential biomarkers for predicting the overall survival

**DOI:** 10.3389/fonc.2022.1050288

**Published:** 2022-12-22

**Authors:** Jing Yang, Zixuan Wu, Xiaoxi Wu, Siya Chen, Xinhua Xia, Jianguo Zeng

**Affiliations:** ^1^Hunan Agricultural University, Changsha, China; ^2^School of Pharmacy, Hunan University of Chinese Medicine, Changsha, China; ^3^The Second Affiliated Hospital of Guangzhou University of Chinese Medicine, Guangzhou, China

**Keywords:** stomach adenocarcinoma (STAD), M6A, predicting model, immunity, SNP, CNV

## Abstract

**Background:**

Stomach adenocarcinoma (STAD) arises from the mutations of stomach cells and has poor overall survival. Chemotherapy is commonly indicated for patients with stomach cancer following surgical resection. The most prevalent alteration that affects cancer growth is N6-methyladenosine methylation (m6A), although the possible function of m6A in STAD prognosis is not recognized.

**Method:**

The research measured predictive FRGs in BLCA samples from the TCGA and GEO datasets. Data on the stemness indices (mRNAsi), gene mutations, copy number variations (CNV), tumor mutation burden (TMB), and corresponding clinical characteristics were obtained from TCGA and GEO. STAD from TCGA and GEO at 24 m6A was investigated. Lasso regression was used to construct the prediction model to assess the m6A prognostic signals in STAD. In addition, the correlation between m6a and immune infiltration in STAD patients was discussed using GSVA and ssGSEA analysis. Based on these genes, GO and KEGG analyses were performed to identify key biological functions and key pathways.

**Result:**

A significant relationship was discovered between numerous m6A clusters and the tumor immune microenvironment, as well as three m6A alteration patterns with different clinical outcomes. Furthermore, GSVA and ssGSEA showed that m6A clusters were significantly associated with immune infiltration in the STAD. The low-m6Ascore group had a lower immunotherapeutic response than the high-m6Ascore group. ICIs therapy was more effective in the group with a higher m6Ascore. Three writers (VIRMA, ZC3H13, and METTL3) showed significantly lower expression, whereas five authors (METTL14, METTL16, WTAP, RBM15, and RBM15B) showed considerably higher expression. Three readers (YTHDC2, YTHDF2, and LRPPRC) had higher levels of expression, whereas eleven readers (YTHDC1, YTHDF1, YTHDF3, HNRNPC, FMR1, HNRNPA2B1, IGFBP1, IGFBP2, IGFBP3, and RBMX) had lower levels. As can be observed, the various types of m6 encoders have varied ramifications for STAD control.

**Conclusion:**

STAD occurrence and progression are linked to m6A-genes. Corresponding prognostic models help forecast the prognosis of STAD patients. m6A-genes and associated immune cell infiltration in the tumor microenvironment (TME) may serve as potential therapeutic targets in STAD, which requires further trials. In addition, the m6a-related gene signature offers a viable alternative to predict bladder cancer, and these m6A-genes show a prospective research area for STAD targeted treatment in the future.

## Introduction

Gastric cancer (GC) is the fifth most frequent cancer and the third major cause of cancer-related deaths globally. Stomach adenocarcinoma (STAD) is the most frequent histologic form of GC with rapid progression, and malignant GC accounts for 95 percent of all gastric tumors. Research has discovered that Helicobacter pylori infection is responsible for 90% of STAD cases (1). Many researchers have proposed that STAD could also be triggered by autoimmunity, bacteria, and their metabolites (2). STAD is now considered a collection of uncommon illnesses that risk human health (3), underlining the timely intervention of STAD (4). Chemotherapy is an important part of tumor treatment, but their cytotoxicity and potential adverse effects after long-term use will pose considerable issues for patients. Recurring usage of chemotherapy drugs is associated with tumor cell resistance, thereby compromising the curative impact (5). Moreover, the lack of precise biomarkers for early tumor diagnosis and restricted preclinical models result in unsatisfactory STAD therapeutic outcomes (6, 7). Therefore, there is a pressing need to identify novel and reliable biomarkers for the early identification and diagnosis of STAD.

There are 172 identified forms of RNA alterations, and the most common chemical modifications are m6A, m1A, and m5C. m6A is one of the most prevalent eukaryotic mRNA modifications (8). The m6A modification involves the methylation of adenosine’s sixth nitrogen atom, with Sadenosylmethionine, a cellular methyltransferase substrate, functioning as the methyl donor (9). Epigenetic regulation of cell development and differentiation has been linked to m6A and post-translational histone changes (10). m6A was the most prevalent internal epistemic RNA modification, and it was implicated with several biological functions such as RNA splicing, export, and translation (11, 12). M^6^A regulators, such as methyltransferases complex (“writers”), signal transducers (“readers”), and demethylases (“erasers”), control the m^6^A alterations (13). Different levels of m^6^A are associated with tumor stem cell self-renewal, cancer cell proliferation, and chemotherapy sensitivity in numerous studies (14, 15). Tumor stem cell self-renewal is stimulated by aberrant m^6^A, which contributes to tumor growth (16). Thus, a novel anticancer method based on restoring RNA methylation equilibrium in tumor cells was created (17, 18).

Numerous studies have shown that m6A regulators play an important role in tumor immune microenvironment control. FTO has a comparable antitumor effect on melanoma as a factor in anti-PD-1 resistance. The researchers expected that combining FTO inhibitors with anti-PD-1 inhibition would aid in the reduction of treatment resistance in melanoma patients. m6A alteration, as a reversible epigenetic change, should be addressed in the context of tumor therapy.

## Materials and methods

We used the approaches proposed by Zi-Xuan Wu, et al. 2021 (19).

### Datasets and PRGs

The TCGA was used to collect STAD gene and clinical data (20). 375 STADs and 32 normal data were registered in the TCGA on September 6, 2022. The GEO was searched for mRNA expression on September 6, 2022. Series: GSE84437. Platform: GPL6947-13512. The GEO was used to maintain 433 STAD cases (21) (**Table 1**). 24 m6A regulators were identified (22) (**Table S1**).

**Table 1:** Patient clinical features.

TCGA		GEO	
Variable	Number of samples	Variable	Number of samples
Gender		Gender	
Male/Female	285/158	Male/Female	296/137
Age at diagnosis		Age at diagnosis	
≤65/>65/NA	197/241/5	≤65/>65	283/150
Grade		Grade	
G1/G2/G3/G4/NA	Unknown	G1/G2/G3/G4/NA	Unknown
Stage		Stage	
I/II/III/IV/NA	59/130/183/44/27	I/II/III/IV/NA	Unknown
T		T	
T1/T2/T3/T4/NA	23/93/198/119/10	T1/T2/T3/T4	11/38/92/292
M		M	
M0/M1/NA	391/30/22	M0/M1/NA	Unknown
N		N	
N0/N1/N2/N3/NA	132/119/85/88/19	N0/N1/N2/N3	80/188/132/33

### DEGs and mutation rates

Perl matched and sorted transcription data to acquire exact mRNA data. The IDs were converted into gene names. FDR<0.05 and |log2FC|≥1 were utilized to assess a substantial change in m6a-genes expression. The relevance of differentially expressed m6a-genes was investigated (DEGs). Data on the simple nucleotide variation (SNP) and copy number variations (CNV) were obtained from TCGA. The R maftools package was used to evaluate and visualize the SNP mutation. The SNP2APA database was created to investigate the impact of SNPs and give OS across various cancer types. The EDGs and GEO data were subsequently merged from TCGA and matched to obtain m6a-genes. We examined this database for particular SNPs with predictive significance in STAD and investigated the connection between these SNPs and critical m6A-genes.

### Tumor m6a classification based on the DEGs

Cluster analysis was performed using the Limma and ConsensusClusterPlus package, and the prognosis-related m6a-genes were separated into three clusters: clusters A, B and C. Survminer was used to study the survivability of m6a-genes, and survival was used to evaluate the predictive utility of m6a-genes. The limma was utilized to discover changes in particular genes between subtypes and tissue types.

### GSEA analysis and cluster DEGs

GSEA was done in GEO and TCGA groups to compare the median level of three clusters’ expression to explore the biological signaling route. NES, gene ratio, and P-value were used to identify KEGG pathways with significant enrichment data. The enriched values (ssGSEA) of immune cells in both the 3 cohorts and the PCA methods were investigated. To test whether or not the prognosis model successfully divided patients into three groups. To assess a substantial change in m6a-genes expression, FDR<0.001 and |log2FC|≥1 were utilized. The relevance of differentially expressed m6a-genes was Cluster DEGs.

### GO and KEGG analysis

Using GO and KEGG, the biological function and pathways linked with the DEGs were investigated. BP, MF, and CC regulated by differentially expressed m6a-genes were explored using R.

### Prognostic signature and cluster

Every STAD patient’s risk score was evaluated. Lasso regression was associated with prognostic genes. These prognostic genes were subjected to cluster analysis to assess their respective expression in different clusters. Accordingly, the m6a-genes associated with prognosis were obtained after matching. M6a-DEGs were classified under two categories: low- or high-risk. Survival curves for the 2 categories were evaluated. To confirm the impact of clinical variables on this model, a nurse independent prediction analysis was performed. Risk and clinical interaction studies were made available.

### Prognostic m6a-genes and immune cell infiltration

Tumor mutation load (TML) is a good predictor of immunotherapy response that count the amount of mutations per DNA megabase and detect alterations such as nucleotide insertions, base substitutions, and deletions. MSI is a molecular tumor characteristic that is distinguished by spontaneous nucleotide loss or gain from short tandem repeat DNA sequences. The association between m6a-genes Expression and TMB and MSI was explored.

## Results

### The m6A regulator landscape

**Figure 1:**
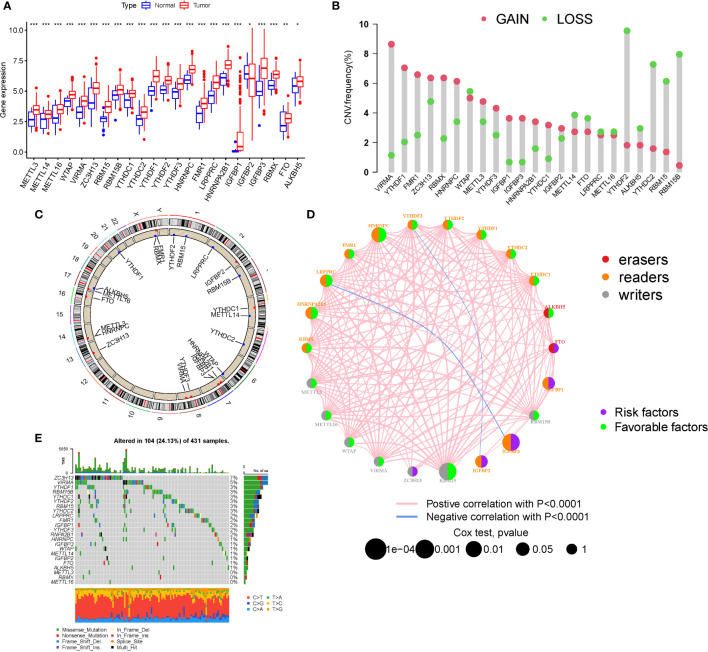
Landscape of genetic and expression variation. **(A)** The expression of m6A regulators. **(B)** The CNV variation frequency. **(C)** CNV Analysis (M6a shows a significant association with CNV in STAD). **(D)** The interaction between m6A regulators (The m6A regulatory network is a study of the expression correlation and prognostic importance of the 24 m6A regulators in STAD patients). **(E)** Waterfall plot of TMB (the frequency of m6A regulator mutations was lower than 7%). (*P < 0.05; **P < 0.01; ***P < 0.001).

M6A regulator mRNA expression changed between STAD and normal tissues (**Figure 1A**). The expression of each eraser (FTO, ALKBH5) was considerably increased in STAD tissues. Three writers (VIRMA, ZC3H13, and METTL3) revealed noticeably reduced expressions, whereas five writers (METTL14, METTL16, WTAP, RBM15, and RBM15B) displayed conspicuously high expressions. Three readers (YTHDC2, YTHDF2, and LRPPRC) were expressed at greater levels, while ten readers (YTHDC1, YTHDF1, YTHDF3, HNRNPC, FMR1, HNRNPA2B1, IGFBP1, IGFBP2, IGFBP3, and RBMX) were expressed at lower levels. The CNVs of the 23 m6A regulators were then summarized in STAD (**Figure 1B**). The results of the SNV analysis are shown as a circle graph (**Figure 1C**; **Table S2**). The m6A regulatory network represents an investigation into the expression correlation and prognostic significance of the 24 m6A regulators in STAD patients (**Figure 1D**; **Table S3**). To determine the hazard ratios (HRs) and PFS for the m6A regulators, a univariate Cox regression model was employed (**Table S1**). Consequently, ZC3H13, FTO, IGFBP1, IGFBP2, and IGFBP3 were identified as risk factors, whereas RBM15, HNRNPC, and HNRNPA2B1 were identified as protective factors. Furthermore, the frequency of m6A regulator mutations was lower than 7% (**Figure 1E**), indicating no relationship to tumor growth. Low expression of erasers, as prognostic variables, revealed that a reduction in m6A demethylation can inhibit the development of STAD. Upregulation of three STAD readers (YTHDC2, YTHDF2, and LRPPRC) is associated with poor prognosis. **Table S2** contains a complete study of RNA expression and CNV.

### Mutation and expression correlation, m6A survival analysis

**Figure 2:**
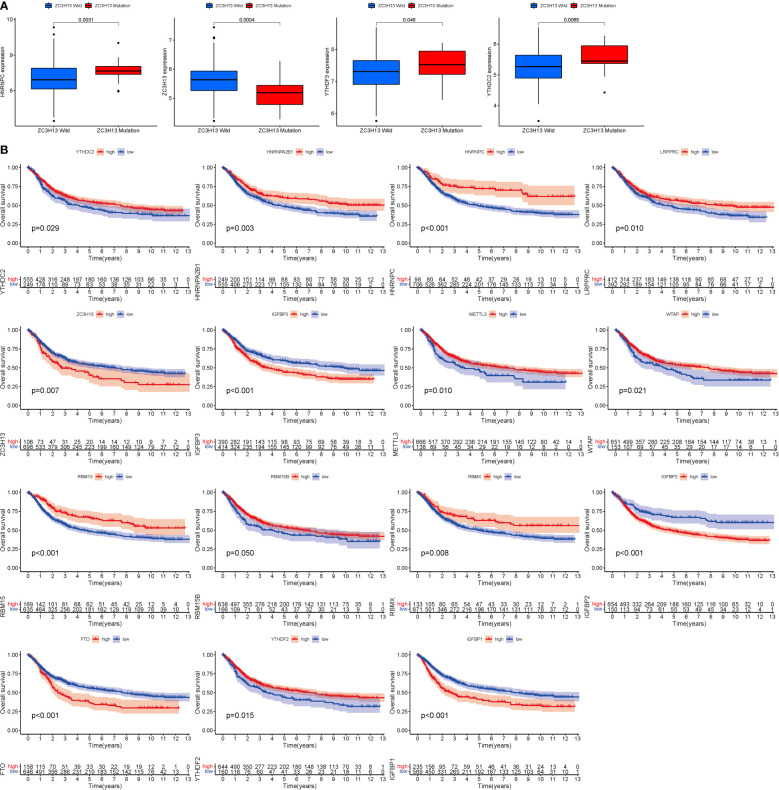
Mutation and expression correlation. **(A)** Mutation and expression (There was considerable variation in four genes (HNRNPC, YTHDC2, YTHDF3, and ZC3H13). **(B)** m6A survival analysis (All of these m6a-genes were associated with STAD prognosis.).

ZC3H13 is the gene with the highest mutation rate (**Figure 1E**), based on which grouping analysis was performed. There was significant variability in four genes (HNRNPC, YTHDC2, YTHDF3 and ZC3H13) (**Figure 2A**). In terms of survival analysis, all these m6a-genes correlated with the prognosis of STAD, suggesting a strong relationship between m6a-genes on the progression of STAD and the survival status of patients (**Figure 2B**).

### Tumor classification and immune cells

**Figure 3:**
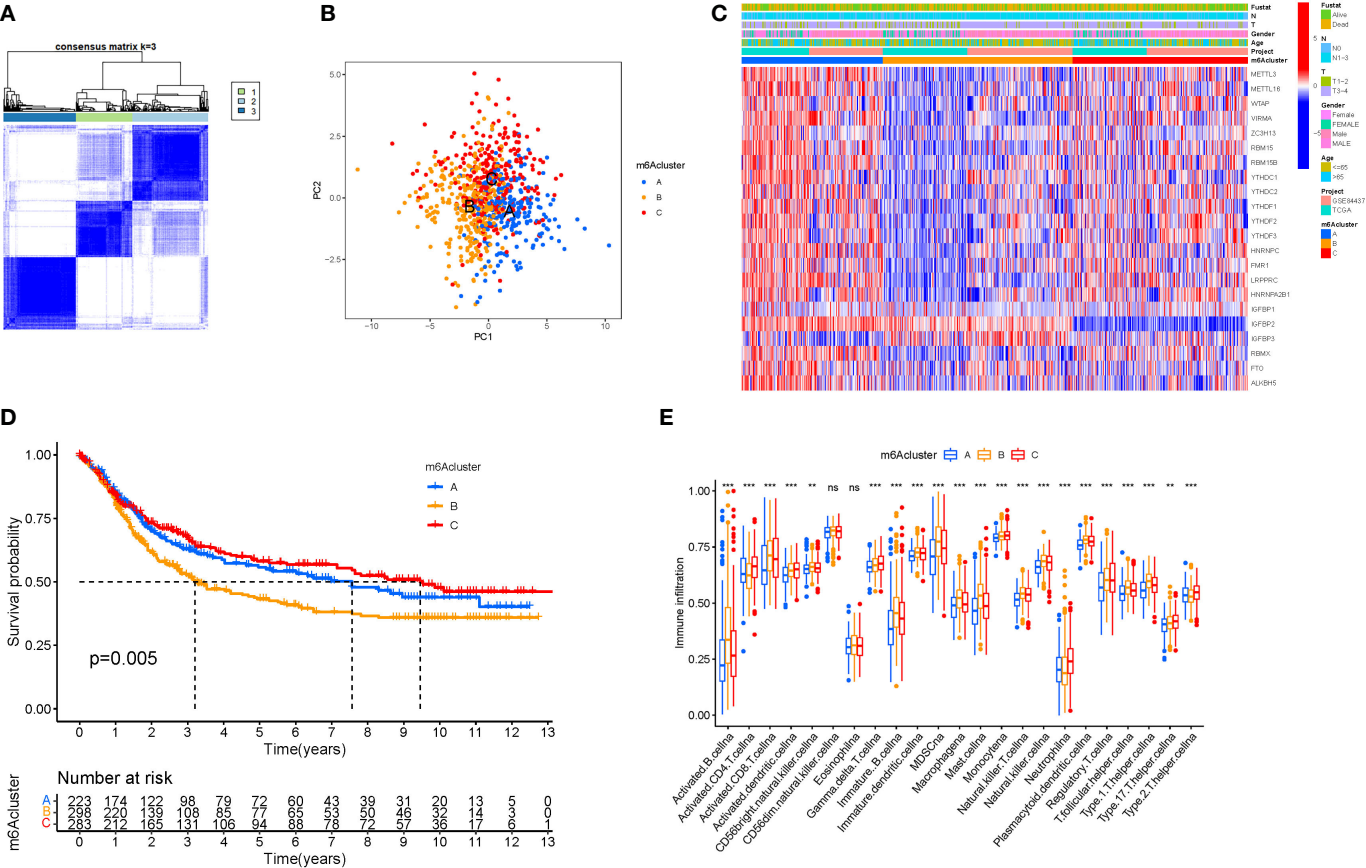
Tumor classification and Immune cells. **(A)** The consensus (When k was 3, intraorganizational ties were the greatest and intergenerational relationships were the lowest). **(B)** PCA (The patients were separated into three groups based on their level of risk). **(C)** Heatmap. **(D)** Kaplan-Meier OS curves (Cluster C had a greater survival rate). **(E)** Enriched values of immune cells (Except for CD56dim.natural.killer.cell and Eosinophil, the variability across the three groups was extremely significant). (na P > 0.05; **P < 0.01; ***P < 0.001).

**Figure 4:**
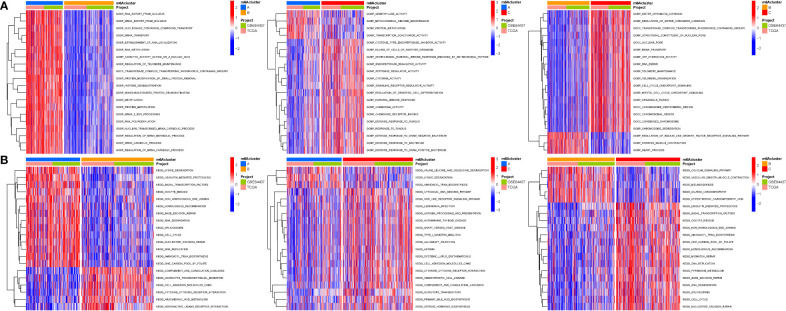
GSEA. **(A)** GO. **(B)** KEGG. These genes were associated with RNA and Methylation.

When k was 3, intraorganizational links were strongest and intergenerational connections were weakest (**Figure 3A**). The PCA efficiently classified STAD’s individuals with various dangers into three basic categories (**Figure 3B**). A heatmap reflects both the m6a genes and clinical characteristics (**Figure 3C**). M6a Clusters were employed in survival research to investigate PRG predictive capacity, and Cluster C had a greater survival rate (P=0.005; **Figure 3D**; **Table S4**). The enriched values of immune cells in both 3 cohorts were studied. The variability between the three groups was highly significant except for two cells(CD56dim.natural.killer.cell and Eosinophil) (**Figure 3E**). The functional enrichment of m6a-genes was determined using GSEA (**Figures 4A, B**). These genes were associated with RNA and Methylation.

### Cluster variance analysis and differential gene identification

**Figure 5:**
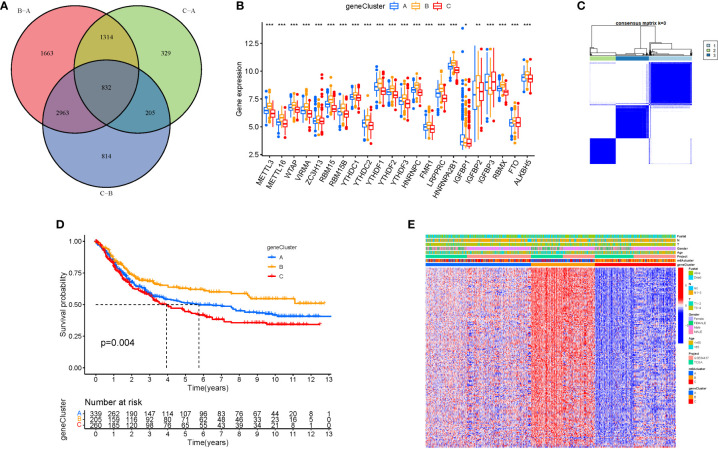
Cluster variance analysis and DEGs. **(A)** VNN (Correlation of differential expression of m6a gene among the three clusters). **(B)** Boxplot. **(C)** The consensus (When k was 3, intraorganizational links were strongest and intergenerational connections were weakest). **(D)** Kaplan-Meier OS curves (Cluster B had a greater survival rate). **(E)** Heatmap.

To further analyse the differential expression of the m6a gene in these clusters, a differential analysis (**Figures 5A, B**) was performed. When k was 3, intraorganizational links were strongest and intergenerational connections were weakest (**Figure 5C**). DEGs were employed in survival research to investigate Cluster DEGs’ predictive potential, and Cluster B had a greater survival rate (P=0.004; **Figure 5D**). A heatmap reflects both the Cluster DEGs and clinical characteristics (**Figure 5E**).

### GO and KEGG analysis

**Figure 6:**
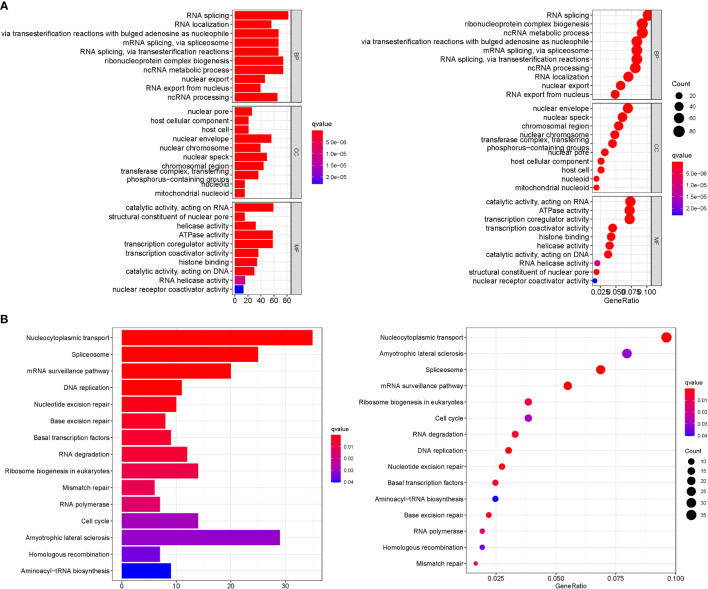
Enrichment analysis. **(A)**: GO. **(B)**: KEGG.

GO analysis revealed 707 core targets. The MF mainly involves signaling transcription coregulator activity (GO:0003712), ATPase activity (GO:0016887), catalytic activity, and acting on RNA (GO:0140098). The CC mainly involves the mitochondrial inner membrane (GO:0005743), mitochondrial matrix (GO:0005759), nuclear envelope (GO:0005635). The BP mainly involves the regulation of cellular amide metabolic process (GO:0034248), LncRNA metabolic process (GO:0034660), organelle fission (GO:0048285). Furthermore, KEGG analysis was used to identify the primary signaling pathways, which indicated that Cluster DEGs were mostly engaged in the Amyotrophic lateral sclerosis (hsa05014), Spliceosome (hsa03040), Cell cycle (hsa04110) (**Figure 6**; **Tables S5, 6**).

### m6A score construction

**Figure 7:**
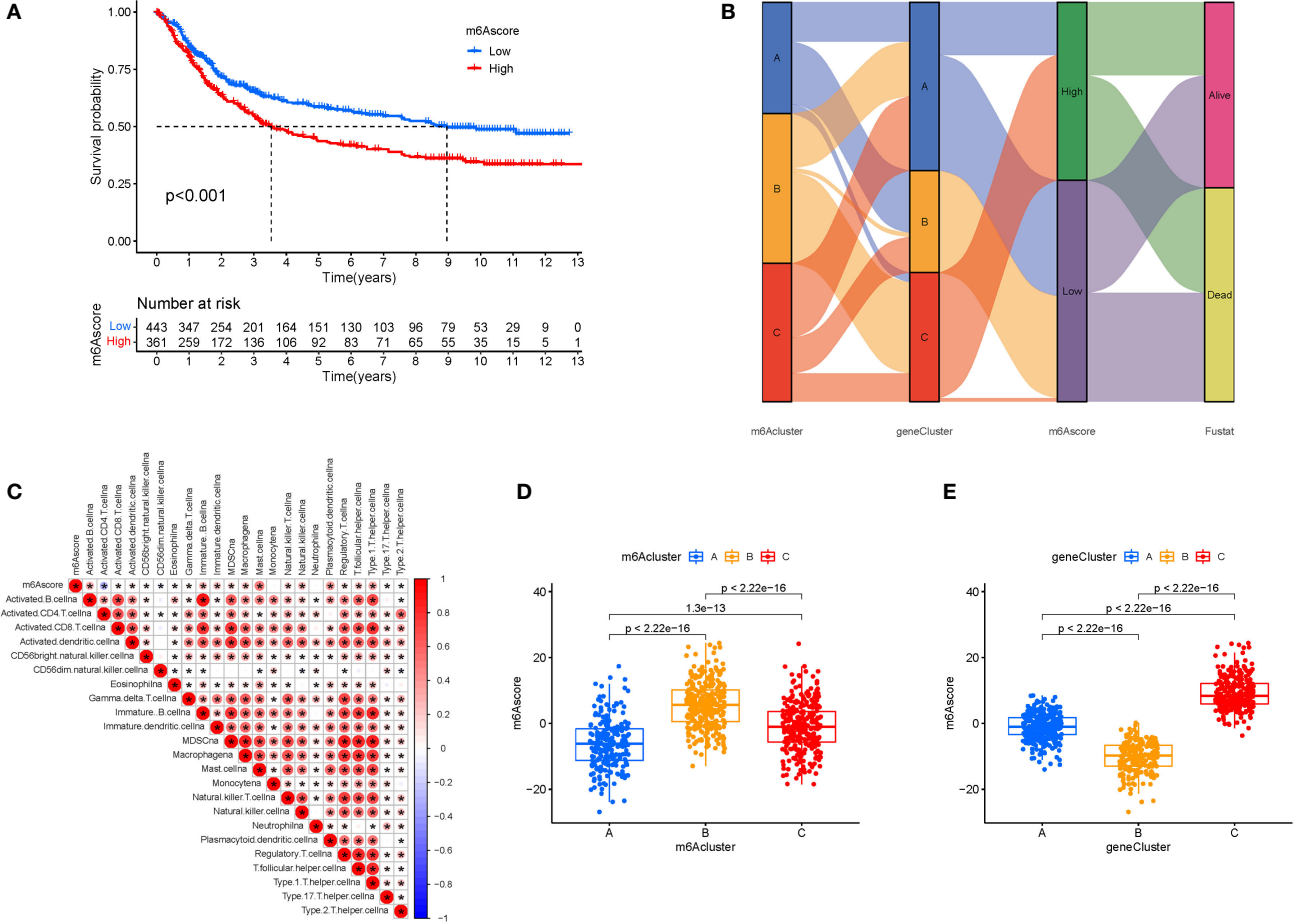
M6A Score Construction. **(A)** Survival (The high-m6Ascore group fared better than the low-m6Ascore group). **(B)** Ggalluvial plot. **(C)** Immune cells and prognosis m6A-genes. **(D)** m6A regulators cluster (The highest m6Ascore was discovered in the m6A regulators cluster B, whereas the lowest m6Ascore was discovered in the m6A regulators cluster A). **(E)** Gene cluster.

We created the m6Ascore, a set of scoring methods for assessing the m6A modification pattern of individual STAD patients, in response to the demand for more reliable and clear models for immunotherapy prediction based on these phenotype-related genes. Although survival analysis indicated two distinct prognoses, the cluster of m6A regulators and CEGs were separated into three groups. As a consequence, CEG PCA was used to distinguish between high and low m6Ascores. The high-m6Ascore group had a better prognosis than the low-m6Ascore group based on the results of the Kaplan-Meier survival analysis (**Figure 7A**). The relationships between m6A regulator clusters, immunological clusters, gene clusters, and m6Ascore groups were studied. It is evident that the relationships between the different m6a and gene clusters are intricate and their correspondence with survival states is not one-to-one. Cluster B has a higher probability of survival state (**Figure 7B**). In addition, as seen in the figure, multiple m6A-genes in STAD were significantly correlated with T and B cells, suggesting that m6A-genes affect immune cells and thus the development of STAD (**Figure 7C**). The greatest m6Ascore was found in m6A regulators cluster B, whereas the lowest m6Ascore was found in m6A regulators cluster A. (**Figure 7D**). Gene cluster C had the highest m6Ascore (**Figure 7E**).

### TMB score construction

**Figure 8:**
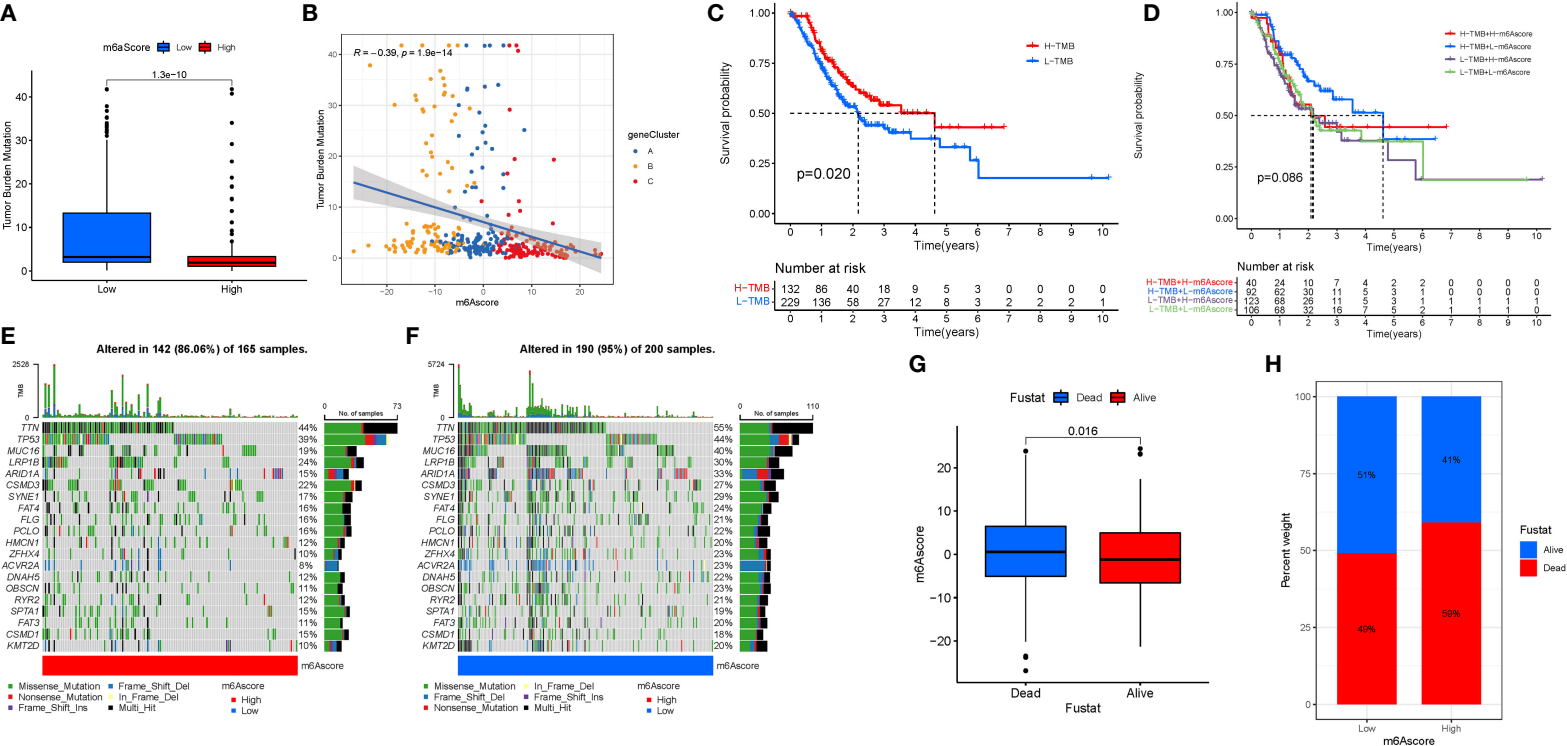
TMB Score Construction. **(A)** TMB of m6Ascore.group. **(B)** Tumor Burden Mutation with m6Ascore. **(C)** TMB.survival. **(D)** TMB-score.survival. **(E)** High mutations. **(F)** Low mutations. **(G, H)** Survival investigation.

We also created the TMBscore, a set of scoring methods for measuring the pattern of gene mutations in specific STAD patients. The greater Tumor Burden Mutation was found in low in m6Ascore.group (**Figure 8A**). Tumor Burden Mutation is negatively correlated with m6Ascore. It can be seen that m6Ascore is not a good predictor of gene mutations in STAD patients (**Figure 8B**). To test the predictability of TMB, a survival investigation was performed, and H-TMB had a greater chance of survival (**Figure 8C**). However, there was no difference in the survival analysis between TMB and m6Ascore (**Figure 8D**). Furthermore, separate analyses were carried out for high and low mutations based on the three groups of mutated genes. The gene mutation status was shown with a waterfall plot. The overall average mutation frequency of DEGs in the prognostic model ranged from 20 to 55% and 10 to 44%, suggesting that STAD mutations might be associated with the deregulation of critical genes (**Figures 8E, F**). The clinical data were incorporated to predict survival for the grouping of mutations. Mortality was higher and significantly different when the m6ascore increased. In addition, according to the risk assessment of survival, the survival rate was 51% in the low risk group compared to 41% in the high scoring risk group (**Figures 8G, H**).

### M6A regulation and immunotherapy

**Figure 9:**
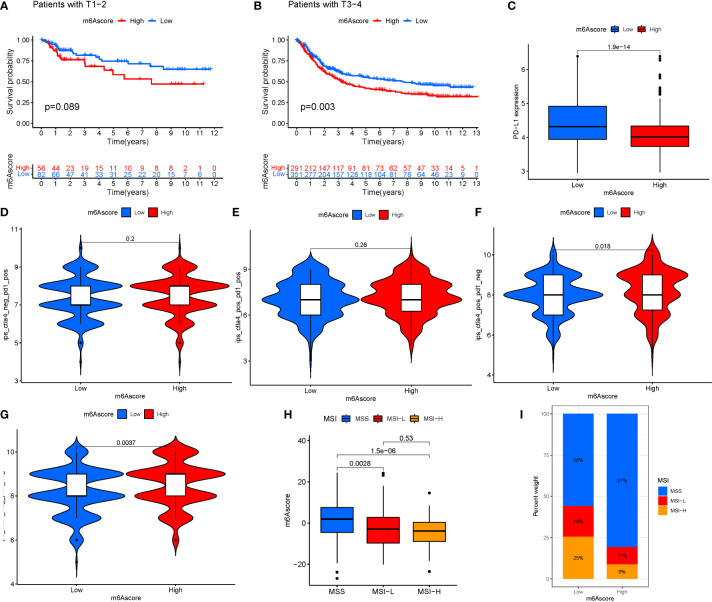
**(A)** T1-T2. **(B)** T3-4. **(C)** Anti-PD-1/L1 immunotherapy. **(D)** ips_ctla4_neg_pd1_neg. **(E)** ips_ctla4_neg_pd1_pos. **(F)** ips_ctla4_pos_pd1_neg. **(G)** ips_ctla4_pos_pd1_pos. **(H, I)** MSI analysis.

Immunotherapy is a significant advancement in cancer treatment. The correlation of m6A modification with the impact of tumor immunotherapy based on the association between m6A alteration and immune cell infiltration in STAD was evaluated. There was no difference in tumor staging between T1-T2, while the difference between T3-4 was significant (**Figures 9A, B**). Anti-PD-1/L1 immunotherapy provided substantial therapeutic benefits and clinical response in individuals with two m6Ascore compared. Furthermore, patients with a low m6Ascore had considerably higher CD274 expression, indicating a possible successful response to anti-PD-1/L1 immunotherapy (**Figure 9C**). Significant difference in the analysis of immunotherapy results when ctla4 is positive and pd1 is negative or ctla4 is negative and pd1 is positive (**Figures 9D–G**). MSI demonstrates that m6Ascore expression is highest in MSS at both high and low risk groups (**Figures 9H, I**). The tumor composed of a complicated TME. New data demonstrates its crucial involvement in tumor development, immune escape, and immunotherapy response. We found several various tumor immune phenotypes in this study by parsing the TME landscape heterogeneity and complexity, which will also increase the capacity of the results of this investigation to advise and forecast immunotherapy reactivity.

## Discussion

The clinical management of STAD is a serious clinical concern due to rapid disease progression and poor prognosis. The level of precision medicine for STAD is hampered by a lack of effective tumor-killing initiators and selective tumor-targeting therapeutic medicines (23). A recent study found that alterations of the mechanism of programmed tumor cell death may potentiate the targeted therapeutic impact of STAD (24). Thus, early identification and diagnosis of the illness are essential. m6A is the most common internal alteration of mRNAs in all higher eukaryotes, and it is methodically regulated by a slew of ‘writers,’ ‘erasers,’ and ‘readers.’ (15) The alteration of m6A affects cancer processing. m6A controls cellular proliferation and maturation, both of which are linked to cancer genesis (13). Evidence shows that abnormal m6A methylation may promote cancer development by upregulating oncogenes and suppressing tumor suppressor genes and that m6A RNA methylation can be altered by the expression of m6A regulators and the activity of m6A enzymes, further impacting tumor progression (25). Based on the interactions of m6A regulators in STAD, the tendencies of m6A readers, writers, and erasers were determined. All erasers’ expression levels were reduced, whereas the majority of readers’ expression levels were considerably increased. This pattern shows that STAD aided in the methylation of m6A RNA. Furthermore, these variables control immune cell infiltration in STAD (26).

Two “writer” genes, METTL3 and METTL14, were shown to have opposing expression patterns in cancers compared to normal indicating that METTL3 and METTL14 functioned as a complex regulator in STAD. Furthermore, the link between m6a-genes, immune cell infiltration and immune checkpoint therapy was investigated. Recent studies have discovered a relationship between RNA modifications mechanisms and anticancer immunity (27). In the last decade, immune checkpoint inhibitors (ICIs) have revolutionized cancer treatment. In ICI-resistant cancers, activation of pyroptosis, ferroptosis, and necroptosis resulted in synergistically improved anticancer efficiency (28). However, there are few studies on the impact of m6a on ICIs. Many patients are resistant to immune checkpoint blockade (ICB) therapy, and multiple pathologic processes are regulated by m6A in RNA (29). According to Na Li’s findings, tumors are more susceptible to cancer immunotherapy in the absence of the m6A demethylase Alkbh5. The inhibitor of Alkbh5 has been shown to improve the effectiveness of cancer immunotherapy (30). Anti-PD-1/L1 immunotherapy offers significant therapeutic benefits and clinical response in persons with two m6Ascores assessed in the present study. Furthermore, patients with a low m6Ascore had significantly increased CD274 expression, indicating that anti-PD-1/L1 immunotherapy might be effective. According to a recent study, METTL3 plays a critical role in several forms of cancer. The abnormal m6A mutation in large intestine cancer was shown to be induced by overexpression of METTL3, which has been linked to tumor spread (31), and METTL3 was downregulated in STAD tissues in this investigation. Moreover, METTL3 has been shown to be oncogenic in the majority of malignancies and to be a tumor suppressor in RCC, bladder cancer, and glioblastoma stem cell (32). Diverse groups have demonstrated that METTL3 has either oncogenic or tumor-suppressive properties, which may be explained by tumor heterogeneity and/or diverse model systems used in the study, and further research is required to gain a better understanding.

KEGG analysis found that the genes were primarily involved in the mRNA surveillance pathway. The NMD mRNA surveillance pathway downregulates aberrant E-cadherin transcripts in gastric cancer cells and in CDH1 mutation carriers. In GSVA, the hedgehog signaling pathway was the most significantly enriched pathway. Smo and Gli1 genes are components of the hedgehog signaling pathway, and their over-expression may trigger STAD. The degree of expression is linked to the stage and severity of STAD (33). Furthermore, studies have shown that Hedgehog-interacting protein (HHIP) may inhibit the growth and proliferation of STAD cell lines by blocking Hedgehog signal transduction, which may serve as a new biological marker for STAD and a new approach for STAD treatment by targeting the drug target of HHIP formation (34). Overactivation of the hedgehog pathway is linked to the occurrence and progression of STAD, and specific targeted therapy targeting this pathway shows good potential as an effective measure for the clinical management of STAD (35).M6A genes may influence STAD cell migration and proliferation *via* modulating the nod like receptor signaling pathway.

This study examined numerous features of STAD using cluster analysis. The expression of 24 m6A regulators was divided into three unique patterns. Interactions between m6A regulators might result in m6A methylation or demethylation. The m6A regulator clusters indicated that tumor cells control their activity by different RNA regulation patterns. Survival disparities in diverse patterns suggest that m6A regulatory patterns have a major influence on STAD patients’ survival. However, classification of m6A regulators into three categories is insufficient to accurately reflect patient prognosis. As a result, the m6Ascore was developed to differentiate the m6A expression patterns and directly predict prognosis. In our investigation, the m6Ascore was linked to the impact of immunotherapy. Individuals with a low m6Ascore had a better prognosis than those who did not get immunotherapy. The higher mutation rate of the low m6Ascore group, along with the release of SNV neoantigens and ITH, might explain this result. The higher the mutation rate in tumor tissue, the more immunogenicity that has been stored is released, and treatment efficacy improves. As a result of the discovery of novel ICIs targeting CTLA4 and PD1, which are generated by activated T cells, neoantigens have emerged as important tumor antigens for the human immune system. However, other researchers observed an inverse association between ITH and the immune checkpoint response in liver cancer. Patients with significant tumor heterogeneity responded favorably to immunotherapy. The reason may be that tumor heterogeneity reacts differently to immunoreactivity, whereas tumor heterogeneity does not necessarily determine immunotherapy response.

The tumor part was composed of a complex TME. As the understanding of the diversity and complexity of the tumor microenvironment deepens, emerging evidence reveals its critical role in the tumor progression, immune escape, and its effect on response to immunotherapy (36). Based on the link between m6A change and immune cell infiltration in STAD, the correlation of m6A modification with the impact of tumor immunotherapy was investigated. Patients with a low m6Ascore had significantly increased CD274 expression, indicating the effectiveness of anti-PD-1/L1 immunotherapy. There is a significant difference in immunotherapy outcomes when ctla4 is positive and pd1 is negative or when ctla4 is negative and pd1 is positive. According to MSI, m6Ascore expression is the greatest in MSS at both high and low risk. Therefore, this study comprehensively through parsing the TME landscape heterogeneity and complexity, we identified some different tumor immune phenotypes, which may also provide benefits to guide and predict the reactivity of immunotherapy.

The relationship between m6a and STAD has been marginally explored. Currently, four papers have used bioinformatics analysis to explore the relationship between m6a and STAD (37–40). Yao Qi et al. observed the expression of METTL14 in STAD by bioinformatics and immunohistochemistry assays. METTL14 expression was substantially downregulated in STAD, reflecting the contribution of major tumors in STAD, progression of TNM staging and poor OS. Furthermore, the inhibitory effects of METTL14 on STAD cell proliferation, migration and invasion have been demonstrated in *in vitro* experiments. Na Luo et al. identified a 10-gene signature based on probable predictive m6a regulatory genes, and these genes (METTL3, WTAP, RBM15, RBM15B, YTHDC2, YTHDF2, HNRNPC, FMR1, LRPPRC, and RBMX) were found to be negatively associated with STAD clinical stage. 23 m6a genes were found in STAD Prognosis by Zhang Meijing et al.

The uniqueness of the study is as follows. First, the current study complemented prior papers with additional FRG data from the TCGA database, which is constantly updated. Second, TCGA data were used as the primary analysis, with GEO data included into the common pattern for model validation. Third, to strengthen the credibility of the results, several databases were employed to measure immune cells and function.

Our analysis has the following limitations: (1) the present study did not obtain sufficient different data sources from other publicly available sites to validate the model’s trustworthiness. (2) The functional enrichment processes at work in the regulatory networks of various risk groups were explored, but their specific mechanisms in permitting m6A require more investigation to confirm the current findings. (3) The prediction model created in this study must be externally and practically validated.

## Conclusions

We discovered three distinct m6A regulatory patterns for STAD, as well as transcriptome and immune infiltration characteristics in distinct m6A regulatory patterns. The current study specifies the functions of m6A regulators and explains the underlying causes of different clinical outcomes and immunotherapy responses in different m6A regulatory patterns. A detailed investigation of individual m6A regulation patterns will contribute to the formulation of personalized immunotherapy treatments for STAD patients and provide a better understanding of STAD immune-cell characterization.

Furthermore, the goal of this work is to discover and thoroughly profile the gene signatures of m6A-related regulators in STAD. The many m6A alteration patterns contributed significantly to the TME’s variety and complexity. A predictive model was also created based on the m6a gene signature, which might predict the clinical course of STAD. Our findings suggest that the m6A genes are promising prognostic markers that might give new insights into STAD treatment options and guide successful immunotherapy.

## Data availability statement

The original contributions presented in the study are included in the article/**Supplementary Material**. Further inquiries can be directed to the corresponding authors.

## Author contributions

JY and ZW drafted and revised the manuscript. JY and XW revised the manuscript. SC and XW were in charge of data collection. JZ and XX conceived and designed this article, in charge of syntax modification and revised of the manuscript. All authors contributed to the article and approved the submitted version.

## Funding

1. Hunan Province Traditional Chinese Medicine Research Program (NO.2021211); 2. First-class Discipline Project on Chinese Pharmacology of Hunan University of Chinese Medicine (No. 201803).

## Acknowledgments

Thanks for your thoughtful remarks, reviewers and editors.

## Conflict of interest

The authors declare that the research was conducted in the absence of any commercial or financial relationships that could be construed as a potential conflict of interest.

## Publisher’s note

All claims expressed in this article are solely those of the authors and do not necessarily represent those of their affiliated organizations, or those of the publisher, the editors and the reviewers. Any product that may be evaluated in this article, or claim that may be made by its manufacturer, is not guaranteed or endorsed by the publisher.
