# Supplementary material for: Constructing and validating of m6a-related genes prognostic signature for stomach adenocarcinoma and immune infiltration: Potential biomarkers for predicting the overall survival
Source: Front Oncol. 2022 Dec 22;12:1050288. doi: 10.3389/fonc.2022.1050288 (PMC9814967; doi:10.3389/fonc.2022.1050288)
Supplement: Supplementary file 1 [file Table_1.doc]

Constructing and Validating of m6a-Related Genes Prognostic Signature for Stomach adenocarcinoma and Immune Infiltration: Potential Biomarkers for Predicting the Overall Survival

**Supplementary appendix to the manuscript**

Contents of supplementary appendix

[Appendix 1 3](#__RefHeading___Toc2666)

[382 ferroptosis-related genes 3](#__RefHeading___Toc22386)

[Table 1a. 150 ferroptosis-related genes of Driver. 3](#__RefHeading___Toc30433)

[Table 1b. 109 ferroptosis-related genes of suppressor. 28](#__RefHeading___Toc26161)

[Table 1c. 123 ferroptosis-related genes of marker. 51](#__RefHeading___Toc19419)

[Appendix 2 67](#__RefHeading___Toc27325)

[DEGs linked to FRGs 67](#__RefHeading___Toc7784)

[Table 2. 208 DEGs linked to FRGs. 67](#__RefHeading___Toc4768)

[Appendix 3 73](#__RefHeading___Toc1980)

[hub genes analysis 73](#__RefHeading___Toc29900)

[Table 3. Hub genes. 73](#__RefHeading___Toc17254)

[Appendix 4 77](#__RefHeading___Toc25961)

[The gene expression profile and clinical characteristics 77](#__RefHeading___Toc10330)

[Table 4. The gene expression profile and clinical characteristics. 77](#__RefHeading___Toc11593)

[Appendix 5 79](#__RefHeading___Toc6954)

[5 risk FRGs 79](#__RefHeading___Toc27053)

[Appendix 6 94](#__RefHeading___Toc19232)

[Table 6. Clinical features for the TCGA cohort. 94](#__RefHeading___Toc16257)

[Appendix 7 110](#__RefHeading___Toc22813)

[GO enrichment analysis 110](#__RefHeading___Toc9537)

[Table 7a. BP of GO enrichment analysis. 110](#__RefHeading___Toc15966)

[Table 7b. CC of GO enrichment analysis. 161](#__RefHeading___Toc24856)

[Table 7c. MF of GO enrichment analysis. 162](#__RefHeading___Toc15012)

[Appendix 8 165](#__RefHeading___Toc1284)

[KEGG enrichment analysis 165](#__RefHeading___Toc3146)

[Table 8. KEGG enrichment analysis. 165](#__RefHeading___Toc16635)

[Appendix 9 168](#__RefHeading___Toc2632)

[gene set enrichment analyses (GSEA) 168](#__RefHeading___Toc25455)

[Table 9a. GSEA of high rish. 168](#__RefHeading___Toc14149)

[Table 9b. GSEA of low rish. 173](#__RefHeading___Toc25239)

# Appendix 1

**M6a gene**

**Table S1. M6a gene.**

| Gene | Type |
| --- | --- |
| METTL3 | writers |
| METTL14 | writers |
| METTL16 | writers |
| WTAP | writers |
| VIRMA | writers |
| ZC3H13 | writers |
| RBM15 | writers |
| RBM15B | writers |
| YTHDC1 | readers |
| YTHDC2 | readers |
| YTHDF1 | readers |
| YTHDF2 | readers |
| YTHDF3 | readers |
| HNRNPC | readers |
| FMR1 | readers |
| LRPPRC | readers |
| HNRNPA2B1 | readers |
| IGFBP1 | readers |
| IGFBP2 | readers |
| IGFBP3 | readers |
| RBMX | readers |
| FTO | erasers |
| ALKBH5 | erasers |

# Appendix 2

## **CNV analysis**

**Table S2a. Rcircos.geneLabel.**

| Chromosome | chromStart | chromEnd | Gene |
| --- | --- | --- | --- |
| chr1 | 28736621 | 28769775 | YTHDF2 |
| chr1 | 110338506 | 110346681 | RBM15 |
| chr2 | 43886508 | 43996005 | LRPPRC |
| chr2 | 216632828 | 216664436 | IGFBP2 |
| chr3 | 51391268 | 51397908 | RBM15B |
| chr4 | 68310387 | 68350089 | YTHDC1 |
| chr4 | 118685368 | 118715433 | METTL14 |
| chr5 | 113513683 | 113595285 | YTHDC2 |
| chr6 | 159725585 | 159756319 | WTAP |
| chr7 | 26189927 | 26201529 | HNRNPA2B1 |
| chr7 | 45888357 | 45893668 | IGFBP1 |
| chr7 | 45912245 | 45921874 | IGFBP3 |
| chrX | 136848004 | 136880764 | RBMX |
| chrX | 147911951 | 147951125 | FMR1 |
| chr8 | 63168553 | 63212786 | YTHDF3 |
| chr8 | 94487693 | 94553529 | VIRMA |
| chr13 | 45954465 | 46052759 | ZC3H13 |
| chr14 | 21209136 | 21269494 | HNRNPC |
| chr14 | 21498133 | 21511375 | METTL3 |
| chr16 | 53703963 | 54121941 | FTO |
| chr17 | 2405562 | 2511891 | METTL16 |
| chr17 | 18183078 | 18209954 | ALKBH5 |
| chr20 | 63195429 | 63216234 | YTHDF1 |

**Table S2b. Rcircos.scatter.**

| chromosome | start | stop | seg.mean |
| --- | --- | --- | --- |
| chr1 | 28736621 | 28769775 | -1 |
| chr1 | 110338506 | 110346681 | -1 |
| chr2 | 43886508 | 43996005 | -1 |
| chr2 | 216632828 | 216664436 | 1 |
| chr3 | 51391268 | 51397908 | -1 |
| chr4 | 68310387 | 68350089 | 1 |
| chr4 | 118685368 | 118715433 | -1 |
| chr5 | 113513683 | 113595285 | -1 |
| chr6 | 159725585 | 159756319 | -1 |
| chr7 | 26189927 | 26201529 | 1 |
| chr7 | 45888357 | 45893668 | 1 |
| chr7 | 45912245 | 45921874 | 1 |
| chrX | 136848004 | 136880764 | 1 |
| chrX | 147911951 | 147951125 | 1 |
| chr8 | 63168553 | 63212786 | 1 |
| chr8 | 94487693 | 94553529 | 1 |
| chr13 | 45954465 | 46052759 | 1 |
| chr14 | 21209136 | 21269494 | 1 |
| chr14 | 21498133 | 21511375 | 1 |
| chr16 | 53703963 | 54121941 | -1 |
| chr17 | 2405562 | 2511891 | -1 |
| chr17 | 18183078 | 18209954 | -1 |
| chr20 | 63195429 | 63216234 | 1 |

# Appendix 3

# **The Network expression**

**Table S3a. The network edge.**

| from | to | cor | pvalue | color | weight |
| --- | --- | --- | --- | --- | --- |
| FTO | ALKBH5 | 0.442382064112328 | 4.82757387483592e-40 | pink | 2.65429238467397 |
| FTO | YTHDC1 | 0.233018809228097 | 2.00157244022943e-11 | pink | 1.39811285536858 |
| FTO | YTHDC2 | 0.230744506849456 | 3.166243960967e-11 | pink | 1.38446704109673 |
| FTO | FMR1 | 0.180048146197831 | 2.57492575039908e-07 | pink | 1.08028887718698 |
| FTO | IGFBP2 | 0.144752464630358 | 3.62702383585712e-05 | pink | 0.86851478778215 |
| FTO | IGFBP3 | 0.184643997903194 | 1.25048409195151e-07 | pink | 1.10786398741916 |
| FTO | RBMX | 0.215394403720716 | 6.17363683202136e-10 | pink | 1.2923664223243 |
| FTO | METTL3 | 0.208215143751338 | 2.3004987595906e-09 | pink | 1.24929086250803 |
| FTO | METTL16 | 0.270227277254054 | 5.484862489961e-15 | pink | 1.62136366352433 |
| FTO | VIRMA | 0.303389425206024 | 1.15181583479606e-18 | pink | 1.82033655123614 |
| FTO | ZC3H13 | 0.143573416626811 | 4.20270129917232e-05 | pink | 0.861440499760868 |
| FTO | RBM15B | 0.318733101668136 | 1.55124834671144e-20 | pink | 1.91239861000882 |
| ALKBH5 | YTHDC1 | 0.229498351109083 | 4.06246943592572e-11 | pink | 1.3769901066545 |
| ALKBH5 | YTHDC2 | 0.215837307625993 | 5.68373207303203e-10 | pink | 1.29502384575596 |
| ALKBH5 | HNRNPC | 0.323855969520951 | 3.47885236704402e-21 | pink | 1.9431358171257 |
| ALKBH5 | LRPPRC | 0.171940397566083 | 8.80792004787548e-07 | pink | 1.0316423853965 |
| ALKBH5 | IGFBP2 | 0.181687650928619 | 1.99421926727919e-07 | pink | 1.09012590557172 |
| ALKBH5 | METTL3 | 0.20929157652722 | 1.89432260657739e-09 | pink | 1.25574945916332 |
| ALKBH5 | METTL16 | 0.428328744106502 | 2.18895541143928e-37 | pink | 2.56997246463901 |
| ALKBH5 | VIRMA | 0.266434755121418 | 1.34576169502423e-14 | pink | 1.59860853072851 |
| ALKBH5 | ZC3H13 | 0.161531711928367 | 3.93428338743984e-06 | pink | 0.969190271570204 |
| ALKBH5 | RBM15 | 0.246368831087197 | 1.22938335436558e-12 | pink | 1.47821298652318 |
| ALKBH5 | RBM15B | 0.33293371380515 | 2.29097708843068e-22 | pink | 1.9976022828309 |
| YTHDC1 | YTHDC2 | 0.290055612810656 | 3.97276930424689e-17 | pink | 1.74033367686394 |
| YTHDC1 | YTHDF1 | 0.276037359817062 | 1.34876889839152e-15 | pink | 1.65622415890237 |
| YTHDC1 | YTHDF2 | 0.325956127444724 | 1.86917024434742e-21 | pink | 1.95573676466835 |
| YTHDC1 | YTHDF3 | 0.261279190660395 | 4.45638949364855e-14 | pink | 1.56767514396237 |
| YTHDC1 | HNRNPC | 0.28729655754972 | 8.07743221474789e-17 | pink | 1.72377934529832 |
| YTHDC1 | FMR1 | 0.366786596841956 | 3.91659999879314e-27 | pink | 2.20071958105174 |
| YTHDC1 | LRPPRC | 0.253913712851833 | 2.35682920089586e-13 | pink | 1.523482277111 |
| YTHDC1 | HNRNPA2B1 | 0.275936285095227 | 1.38248794152794e-15 | pink | 1.65561771057136 |
| YTHDC1 | RBMX | 0.1679503867272 | 1.58035496009248e-06 | pink | 1.0077023203632 |
| YTHDC1 | METTL3 | 0.322591506920506 | 5.04487270360821e-21 | pink | 1.93554904152304 |
| YTHDC1 | METTL16 | 0.326873835644243 | 1.42263748434924e-21 | pink | 1.96124301386546 |
| YTHDC1 | WTAP | 0.280843758109765 | 4.11936121951944e-16 | pink | 1.68506254865859 |
| YTHDC1 | VIRMA | 0.216641997484035 | 4.88873865463449e-10 | pink | 1.29985198490421 |
| YTHDC1 | ZC3H13 | 0.276245502926865 | 1.28185815654015e-15 | pink | 1.65747301756119 |
| YTHDC1 | RBM15 | 0.431360634510439 | 5.99282074713379e-38 | pink | 2.58816380706263 |
| YTHDC1 | RBM15B | 0.155336966483933 | 9.18070714084779e-06 | pink | 0.932021798903597 |
| YTHDC2 | YTHDF2 | 0.310269515403943 | 1.72240285968835e-19 | pink | 1.86161709242366 |
| YTHDC2 | YTHDF3 | 0.399830672866211 | 2.27378812932193e-32 | pink | 2.39898403719726 |
| YTHDC2 | HNRNPC | 0.308512003313506 | 2.81208010375142e-19 | pink | 1.85107201988104 |
| YTHDC2 | FMR1 | 0.193923305332402 | 2.75027303252849e-08 | pink | 1.16353983199441 |
| YTHDC2 | LRPPRC | 0.288632868151671 | 5.73370060902738e-17 | pink | 1.73179720891003 |
| YTHDC2 | HNRNPA2B1 | 0.243631889803386 | 2.20834065508639e-12 | pink | 1.46179133882032 |
| YTHDC2 | RBMX | 0.248675038041724 | 7.4633322610971e-13 | pink | 1.49205022825035 |
| YTHDC2 | METTL3 | 0.375864391840099 | 1.63605943971088e-28 | pink | 2.25518635104059 |
| YTHDC2 | METTL16 | 0.319935723697444 | 1.09493666601678e-20 | pink | 1.91961434218466 |
| YTHDC2 | WTAP | 0.32296424168793 | 4.52218614926593e-21 | pink | 1.93778545012758 |
| YTHDC2 | VIRMA | 0.229390274319294 | 4.15095978626734e-11 | pink | 1.37634164591576 |
| YTHDC2 | RBM15 | 0.286732511889578 | 9.32949094805709e-17 | pink | 1.72039507133747 |
| YTHDC2 | RBM15B | 0.184004088371579 | 1.3843033107841e-07 | pink | 1.10402453022947 |
| YTHDF1 | YTHDF2 | 0.351008677377427 | 7.71041953192338e-25 | pink | 2.10605206426456 |
| YTHDF1 | YTHDF3 | 0.187084163647225 | 8.45843113276202e-08 | pink | 1.12250498188335 |
| YTHDF1 | FMR1 | 0.250645081151181 | 4.85312435459538e-13 | pink | 1.50387048690708 |
| YTHDF1 | LRPPRC | 0.264664619457276 | 2.03606787363998e-14 | pink | 1.58798771674366 |
| YTHDF1 | HNRNPA2B1 | 0.408687966726502 | 7.06494510976337e-34 | pink | 2.45212780035901 |
| YTHDF1 | IGFBP1 | 0.142983477981415 | 4.52224369368393e-05 | pink | 0.85790086788849 |
| YTHDF1 | RBMX | 0.303393888301364 | 1.15041566849137e-18 | pink | 1.82036332980818 |
| YTHDF1 | METTL3 | 0.293749768650606 | 1.51748957403956e-17 | pink | 1.76249861190364 |
| YTHDF1 | METTL16 | 0.240100905932016 | 4.65245083222288e-12 | pink | 1.4406054355921 |
| YTHDF1 | WTAP | 0.175581209119813 | 5.10577385986491e-07 | pink | 1.05348725471888 |
| YTHDF1 | VIRMA | 0.308119746357138 | 3.13579152356067e-19 | pink | 1.84871847814283 |
| YTHDF1 | RBM15 | 0.45613973874883 | 9.11467472132476e-43 | pink | 2.73683843249298 |
| YTHDF1 | RBM15B | 0.339214840073711 | 3.30421544048498e-23 | pink | 2.03528904044227 |
| YTHDF2 | YTHDF3 | 0.494413833120525 | 4.94452263075411e-51 | pink | 2.96648299872315 |
| YTHDF2 | HNRNPC | 0.348423334626679 | 1.78160760979394e-24 | pink | 2.09054000776007 |
| YTHDF2 | FMR1 | 0.238378087432427 | 6.66377558994174e-12 | pink | 1.43026852459456 |
| YTHDF2 | LRPPRC | 0.28112048769216 | 3.84471168824827e-16 | pink | 1.68672292615296 |
| YTHDF2 | HNRNPA2B1 | 0.532222595809221 | 2.70221021895332e-60 | pink | 3.19333557485533 |
| YTHDF2 | RBMX | 0.462128832919164 | 5.42589594332606e-44 | pink | 2.77277299751498 |
| YTHDF2 | METTL3 | 0.360042129235555 | 3.88469793463992e-26 | pink | 2.16025277541333 |
| YTHDF2 | METTL16 | 0.228213725273766 | 5.24471480035919e-11 | pink | 1.3692823516426 |
| YTHDF2 | WTAP | 0.504161044791558 | 2.60857120624023e-53 | pink | 3.02496626874935 |
| YTHDF2 | VIRMA | 0.174789857054356 | 5.75378095037221e-07 | pink | 1.04873914232614 |
| YTHDF2 | RBM15 | 0.497966123549004 | 7.45648594833131e-52 | pink | 2.98779674129403 |
| YTHDF2 | RBM15B | 0.313744921678267 | 6.47006556239821e-20 | pink | 1.8824695300696 |
| YTHDF3 | HNRNPC | 0.241915853786016 | 3.1767418221346e-12 | pink | 1.4514951227161 |
| YTHDF3 | FMR1 | 0.2521216406928 | 3.50645643010199e-13 | pink | 1.5127298441568 |
| YTHDF3 | LRPPRC | 0.317739149928 | 2.06635658935979e-20 | pink | 1.906434899568 |
| YTHDF3 | HNRNPA2B1 | 0.411275483133766 | 2.51213539225461e-34 | pink | 2.4676528988026 |
| YTHDF3 | IGFBP2 | -0.140737328106575 | 5.96201512738204e-05 | #6495ED | 0.844423968639447 |
| YTHDF3 | RBMX | 0.243545866236947 | 2.24911242380473e-12 | pink | 1.46127519742168 |
| YTHDF3 | METTL3 | 0.28730602791733 | 8.05788959693517e-17 | pink | 1.72383616750398 |
| YTHDF3 | METTL16 | 0.281727198936078 | 3.30405357618732e-16 | pink | 1.69036319361647 |
| YTHDF3 | WTAP | 0.524343429346221 | 2.87921595568795e-58 | pink | 3.14606057607733 |
| YTHDF3 | VIRMA | 0.394761204057434 | 1.58258012677282e-31 | pink | 2.36856722434461 |
| YTHDF3 | RBM15 | 0.427928497105146 | 2.59466648215773e-37 | pink | 2.56757098263088 |
| YTHDF3 | RBM15B | 0.175460084936884 | 5.20019028602986e-07 | pink | 1.0527605096213 |
| HNRNPC | FMR1 | 0.252240395596267 | 3.41567362393944e-13 | pink | 1.5134423735776 |
| HNRNPC | LRPPRC | 0.457284851047852 | 5.33762850396605e-43 | pink | 2.74370910628711 |
| HNRNPC | HNRNPA2B1 | 0.291297729902989 | 2.87899180453159e-17 | pink | 1.74778637941793 |
| HNRNPC | RBMX | 0.428222887963455 | 2.2896904081823e-37 | pink | 2.56933732778073 |
| HNRNPC | METTL3 | 0.359323951303275 | 4.94386021097622e-26 | pink | 2.15594370781965 |
| HNRNPC | METTL16 | 0.32250830991591 | 5.16944455250646e-21 | pink | 1.93504985949546 |
| HNRNPC | WTAP | 0.286041946742136 | 1.11246934132724e-16 | pink | 1.71625168045281 |
| HNRNPC | VIRMA | 0.277528593233124 | 9.35842097632483e-16 | pink | 1.66517155939874 |
| HNRNPC | RBM15 | 0.406817857420951 | 1.48327843373147e-33 | pink | 2.44090714452571 |
| HNRNPC | RBM15B | 0.237833411177829 | 7.46104814672439e-12 | pink | 1.42700046706698 |
| FMR1 | LRPPRC | 0.30276844339059 | 1.36393585306697e-18 | pink | 1.81661066034354 |
| FMR1 | HNRNPA2B1 | 0.267161255050713 | 1.13442559220563e-14 | pink | 1.60296753030428 |
| FMR1 | RBMX | 0.214835903046531 | 6.85030579139509e-10 | pink | 1.28901541827918 |
| FMR1 | METTL3 | 0.193101839036412 | 3.15443897648913e-08 | pink | 1.15861103421847 |
| FMR1 | METTL16 | 0.230046726925663 | 3.64110646339177e-11 | pink | 1.38028036155398 |
| FMR1 | WTAP | 0.255307992118187 | 1.72645459443248e-13 | pink | 1.53184795270912 |
| FMR1 | VIRMA | 0.263740906881834 | 2.52405023437678e-14 | pink | 1.58244544129101 |
| FMR1 | ZC3H13 | 0.22702054778373 | 6.63990298065798e-11 | pink | 1.36212328670238 |
| FMR1 | RBM15 | 0.3176078769361 | 2.14593434876505e-20 | pink | 1.9056472616166 |
| FMR1 | RBM15B | 0.174860575289494 | 5.69279531152817e-07 | pink | 1.04916345173696 |
| LRPPRC | HNRNPA2B1 | 0.274599528156986 | 1.91456249696677e-15 | pink | 1.64759716894192 |
| LRPPRC | IGFBP3 | -0.18373682203746 | 1.44419765280414e-07 | #6495ED | 1.10242093222476 |
| LRPPRC | RBMX | 0.412894883062943 | 1.30913809039583e-34 | pink | 2.47736929837766 |
| LRPPRC | METTL3 | 0.365015876433472 | 7.19161607369982e-27 | pink | 2.19009525860083 |
| LRPPRC | METTL16 | 0.306589871230996 | 4.78873518339522e-19 | pink | 1.83953922738597 |
| LRPPRC | WTAP | 0.216463365074593 | 5.05527952372507e-10 | pink | 1.29878019044756 |
| LRPPRC | VIRMA | 0.498071252421238 | 7.04809788840569e-52 | pink | 2.98842751452743 |
| LRPPRC | RBM15 | 0.386420731592916 | 3.58222611392944e-30 | pink | 2.31852438955749 |
| LRPPRC | RBM15B | 0.239843089766905 | 4.91032254047931e-12 | pink | 1.43905853860143 |
| HNRNPA2B1 | RBMX | 0.438489108898247 | 2.70488513535946e-39 | pink | 2.63093465338948 |
| HNRNPA2B1 | METTL3 | 0.409903002768226 | 4.35239925591138e-34 | pink | 2.45941801660935 |
| HNRNPA2B1 | METTL16 | 0.207530185018072 | 2.60178701161348e-09 | pink | 1.24518111010843 |
| HNRNPA2B1 | WTAP | 0.415576916397248 | 4.4133112795743e-35 | pink | 2.49346149838349 |
| HNRNPA2B1 | VIRMA | 0.207087771048553 | 2.81642099238665e-09 | pink | 1.24252662629132 |
| HNRNPA2B1 | ZC3H13 | 0.229327538815692 | 4.20318651142828e-11 | pink | 1.37596523289415 |
| HNRNPA2B1 | RBM15 | 0.522166650721647 | 1.02349655836238e-57 | pink | 3.13299990432988 |
| HNRNPA2B1 | RBM15B | 0.232071490351032 | 2.42430735019717e-11 | pink | 1.39242894210619 |
| RBMX | METTL3 | 0.463294923326596 | 3.11218742440741e-44 | pink | 2.77976953995957 |
| RBMX | METTL16 | 0.247334724588907 | 9.98100297437335e-13 | pink | 1.48400834753344 |
| RBMX | WTAP | 0.377719925949393 | 8.44248216952711e-29 | pink | 2.26631955569636 |
| RBMX | VIRMA | 0.452301166335561 | 5.39934608258975e-42 | pink | 2.71380699801336 |
| RBMX | RBM15 | 0.293782877130809 | 1.50436068063387e-17 | pink | 1.76269726278486 |
| RBMX | RBM15B | 0.381630694442415 | 2.06431113935332e-29 | pink | 2.28978416665449 |
| METTL3 | METTL16 | 0.339971365022226 | 2.60896615351012e-23 | pink | 2.03982819013335 |
| METTL3 | WTAP | 0.311061509582331 | 1.3795205771863e-19 | pink | 1.86636905749399 |
| METTL3 | VIRMA | 0.351051571873573 | 7.60351768635925e-25 | pink | 2.10630943124144 |
| METTL3 | ZC3H13 | 0.179720504864601 | 2.70909715405832e-07 | pink | 1.07832302918761 |
| METTL3 | RBM15 | 0.28192059849592 | 3.14798600052447e-16 | pink | 1.69152359097552 |
| METTL3 | RBM15B | 0.380178248813483 | 3.49082412744927e-29 | pink | 2.2810694928809 |
| METTL16 | WTAP | 0.141678650914399 | 5.31257656568727e-05 | pink | 0.850071905486396 |
| METTL16 | VIRMA | 0.357957890175846 | 7.80725287500793e-26 | pink | 2.14774734105508 |
| METTL16 | RBM15 | 0.468609435248524 | 2.40413464862391e-45 | pink | 2.81165661149114 |
| METTL16 | RBM15B | 0.256162855913458 | 1.42519180478888e-13 | pink | 1.53697713548075 |
| WTAP | VIRMA | 0.163643743830695 | 2.92547330680781e-06 | pink | 0.981862462984167 |
| WTAP | RBM15 | 0.324285439592605 | 3.06505438569239e-21 | pink | 1.94571263755563 |
| VIRMA | RBM15 | 0.359096583317593 | 5.33531777343028e-26 | pink | 2.15457949990556 |
| VIRMA | RBM15B | 0.31121956006112 | 1.3196363663108e-19 | pink | 1.86731736036672 |
| ZC3H13 | RBM15B | 0.237931964139027 | 7.31018651473696e-12 | pink | 1.42759178483416 |
| RBM15 | RBM15B | 0.183472987009931 | 1.50577275333897e-07 | pink | 1.10083792205958 |

**Table S3b. The network node.**

| id | group | color | shape | frame | pvalue | size |
| --- | --- | --- | --- | --- | --- | --- |
| FTO | erasers | #E41A1C | circle | purple | 0.0876559433524137 | 8 |
| ALKBH5 | erasers | #E41A1C | circle | green | 0.934260358657281 | 8 |
| YTHDC1 | readers | #FF7F00 | circle | green | 0.628061422453021 | 8 |
| YTHDC2 | readers | #FF7F00 | circle | green | 0.0868624325181248 | 8 |
| YTHDF1 | readers | #FF7F00 | circle | green | 0.739652276962005 | 8 |
| YTHDF2 | readers | #FF7F00 | circle | green | 0.139571343838162 | 8 |
| YTHDF3 | readers | #FF7F00 | circle | green | 0.746781149484089 | 8 |
| HNRNPC | readers | #FF7F00 | circle | green | 0.00118426031786063 | 12 |
| FMR1 | readers | #FF7F00 | circle | green | 0.438831685331979 | 8 |
| LRPPRC | readers | #FF7F00 | circle | green | 0.048938449501214 | 10 |
| HNRNPA2B1 | readers | #FF7F00 | circle | green | 0.011120538734732 | 10 |
| IGFBP1 | readers | #FF7F00 | circle | purple | 0.0133240071224321 | 10 |
| IGFBP2 | readers | #FF7F00 | circle | purple | 0.0196004093318138 | 10 |
| IGFBP3 | readers | #FF7F00 | circle | purple | 0.000959330851786806 | 14 |
| RBMX | readers | #FF7F00 | circle | green | 0.0601200879860111 | 8 |
| METTL3 | writers | #999999 | circle | green | 0.274861018486021 | 8 |
| METTL16 | writers | #999999 | circle | green | 0.533196743006175 | 8 |
| WTAP | writers | #999999 | circle | green | 0.164739675741995 | 8 |
| VIRMA | writers | #999999 | circle | green | 0.943928670935287 | 8 |
| ZC3H13 | writers | #999999 | circle | purple | 0.222657977761862 | 8 |
| RBM15 | writers | #999999 | circle | green | 0.000592673622162507 | 14 |
| RBM15B | writers | #999999 | circle | green | 0.28717430876274 | 8 |

# Appendix 4

## **m6A gene survival expression**

**Table S4. m6A gene survival expression.**

| id | HR | HR.95L | HR.95H | pvalue | km |
| --- | --- | --- | --- | --- | --- |
| METTL3 | 0.885604123722192 | 0.712094714807016 | 1.10139093528633 | 0.274861018486021 | 0.00983613646015213 |
| METTL16 | 0.930987829076346 | 0.743468349225437 | 1.16580394954442 | 0.533196743006175 | 0.102753176741648 |
| WTAP | 0.833456740960494 | 0.644568126363585 | 1.07769855604162 | 0.164739675741995 | 0.0209772107581407 |
| VIRMA | 0.992547034322993 | 0.805775517191685 | 1.22261050916122 | 0.943928670935287 | 0.239566965168499 |
| ZC3H13 | 1.1305387879961 | 0.9282076281774 | 1.37697419452733 | 0.222657977761862 | 0.00662353180593001 |
| RBM15 | 0.699185125088247 | 0.570055567057565 | 0.857565239943181 | 0.000592673622162507 | 0.000360258906707656 |
| RBM15B | 0.899238132122207 | 0.739496047883361 | 1.09348686930397 | 0.28717430876274 | 0.0496362335178708 |
| YTHDC1 | 0.934929216431486 | 0.712128753643966 | 1.22743623995585 | 0.628061422453021 | 0.215819138684765 |
| YTHDC2 | 0.84015675874524 | 0.688294802866302 | 1.02552478433056 | 0.0868624325181248 | 0.0289318040676433 |
| YTHDF1 | 0.969777322037257 | 0.809216166077166 | 1.16219632499046 | 0.739652276962005 | 0.128553272518451 |
| YTHDF2 | 0.843257096968868 | 0.672567017787572 | 1.05726643261141 | 0.139571343838162 | 0.0147716796141126 |
| YTHDF3 | 0.968657396781073 | 0.798401761975347 | 1.17521929062033 | 0.746781149484089 | 0.281872614258234 |
| HNRNPC | 0.67925796585394 | 0.537663860376562 | 0.858140965347549 | 0.00118426031786063 | 0.000482331712209905 |
| FMR1 | 0.913030603683844 | 0.7251800690685 | 1.14954191216822 | 0.438831685331979 | 0.060398991030336 |
| LRPPRC | 0.840552577458618 | 0.707099961110485 | 0.999192015740941 | 0.048938449501214 | 0.0101265945858949 |
| HNRNPA2B1 | 0.737748076248631 | 0.583359300070573 | 0.932996566511775 | 0.011120538734732 | 0.00283862192002027 |
| IGFBP1 | 1.09751505117666 | 1.01955133904788 | 1.18144054293938 | 0.0133240071224321 | 1.22826725652558e-05 |
| IGFBP2 | 1.07872294210251 | 1.01221537685877 | 1.14960038389205 | 0.0196004093318138 | 0.000247199691405409 |
| IGFBP3 | 1.17044558343466 | 1.06606186689083 | 1.28505005790812 | 0.000959330851786806 | 0.000189628533801223 |
| RBMX | 0.792032914482459 | 0.621118768882613 | 1.00997775152104 | 0.0601200879860111 | 0.00827730773638746 |
| FTO | 1.18795577687521 | 0.974900207353334 | 1.44757270248452 | 0.0876559433524137 | 0.000298637527048951 |
| ALKBH5 | 0.990466901699816 | 0.788846596465686 | 1.24361908609123 | 0.934260358657281 | 0.113424258442059 |

# Appendix 5

## **GO enrichment analysis**

**Table S5a. BP of GO enrichment analysis.**

| ONTOLOGY | ID | Description | GeneRatio | BgRatio | pvalue | qvalue | Count |
| --- | --- | --- | --- | --- | --- | --- | --- |
| BP | GO:0008380 | RNA splicing | 82/799 | 481/18862 | 9.27E-28 | 3.12E-24 | 82 |
| BP | GO:0006403 | RNA localization | 56/799 | 234/18862 | 8.43E-27 | 1.42E-23 | 56 |
| BP | GO:0000377 | RNA splicing, via transesterification reactions with bulged adenosine as nucleophile | 67/799 | 383/18862 | 1.82E-23 | 1.53E-20 | 67 |
| BP | GO:0000398 | mRNA splicing, via spliceosome | 67/799 | 383/18862 | 1.82E-23 | 1.53E-20 | 67 |
| BP | GO:0000375 | RNA splicing, via transesterification reactions | 67/799 | 386/18862 | 2.87E-23 | 1.93E-20 | 67 |
| BP | GO:0022613 | ribonucleoprotein complex biogenesis | 74/799 | 473/18862 | 9.02E-23 | 5.07E-20 | 74 |
| BP | GO:0034660 | ncRNA metabolic process | 74/799 | 492/18862 | 1.00E-21 | 4.83E-19 | 74 |
| BP | GO:0051168 | nuclear export | 46/799 | 201/18862 | 2.36E-21 | 9.10E-19 | 46 |
| BP | GO:0006405 | RNA export from nucleus | 39/799 | 142/18862 | 2.43E-21 | 9.10E-19 | 39 |
| BP | GO:0034470 | ncRNA processing | 65/799 | 400/18862 | 5.23E-21 | 1.76E-18 | 65 |
| BP | GO:0018205 | peptidyl-lysine modification | 64/799 | 391/18862 | 7.18E-21 | 2.20E-18 | 64 |
| BP | GO:0050657 | nucleic acid transport | 45/799 | 198/18862 | 8.73E-21 | 2.26E-18 | 45 |
| BP | GO:0050658 | RNA transport | 45/799 | 198/18862 | 8.73E-21 | 2.26E-18 | 45 |
| BP | GO:0006611 | protein export from nucleus | 43/799 | 183/18862 | 1.63E-20 | 3.74E-18 | 43 |
| BP | GO:0051236 | establishment of RNA localization | 45/799 | 201/18862 | 1.66E-20 | 3.74E-18 | 45 |
| BP | GO:0051028 | mRNA transport | 39/799 | 152/18862 | 3.51E-20 | 7.39E-18 | 39 |
| BP | GO:0071426 | ribonucleoprotein complex export from nucleus | 36/799 | 130/18862 | 6.27E-20 | 1.24E-17 | 36 |
| BP | GO:0006913 | nucleocytoplasmic transport | 58/799 | 340/18862 | 6.73E-20 | 1.26E-17 | 58 |
| BP | GO:0071166 | ribonucleoprotein complex localization | 36/799 | 131/18862 | 8.30E-20 | 1.47E-17 | 36 |
| BP | GO:0051169 | nuclear transport | 58/799 | 343/18862 | 1.04E-19 | 1.76E-17 | 58 |
| BP | GO:0097064 | ncRNA export from nucleus | 20/799 | 38/18862 | 4.48E-18 | 7.20E-16 | 20 |
| BP | GO:0006409 | tRNA export from nucleus | 19/799 | 34/18862 | 6.76E-18 | 9.90E-16 | 19 |
| BP | GO:0071431 | tRNA-containing ribonucleoprotein complex export from nucleus | 19/799 | 34/18862 | 6.76E-18 | 9.90E-16 | 19 |
| BP | GO:0006406 | mRNA export from nucleus | 31/799 | 113/18862 | 3.24E-17 | 4.37E-15 | 31 |
| BP | GO:0071427 | mRNA-containing ribonucleoprotein complex export from nucleus | 31/799 | 113/18862 | 3.24E-17 | 4.37E-15 | 31 |
| BP | GO:0042254 | ribosome biogenesis | 51/799 | 307/18862 | 3.94E-17 | 4.92E-15 | 51 |
| BP | GO:0015931 | nucleobase-containing compound transport | 46/799 | 253/18862 | 3.94E-17 | 4.92E-15 | 46 |
| BP | GO:0051031 | tRNA transport | 19/799 | 37/18862 | 5.71E-17 | 6.87E-15 | 19 |
| BP | GO:1903311 | regulation of mRNA metabolic process | 53/799 | 334/18862 | 7.21E-17 | 8.38E-15 | 53 |
| BP | GO:0016925 | protein sumoylation | 25/799 | 81/18862 | 1.81E-15 | 2.03E-13 | 25 |
| BP | GO:0016569 | covalent chromatin modification | 61/799 | 461/18862 | 2.21E-15 | 2.40E-13 | 61 |
| BP | GO:0075733 | intracellular transport of virus | 21/799 | 57/18862 | 5.71E-15 | 6.01E-13 | 21 |
| BP | GO:0016570 | histone modification | 59/799 | 448/18862 | 8.03E-15 | 8.20E-13 | 59 |
| BP | GO:0046794 | transport of virus | 21/799 | 64/18862 | 8.31E-14 | 8.23E-12 | 21 |
| BP | GO:0050684 | regulation of mRNA processing | 30/799 | 139/18862 | 1.18E-13 | 1.13E-11 | 30 |
| BP | GO:0044766 | multi-organism transport | 21/799 | 66/18862 | 1.66E-13 | 1.51E-11 | 21 |
| BP | GO:1902579 | multi-organism localization | 21/799 | 66/18862 | 1.66E-13 | 1.51E-11 | 21 |
| BP | GO:0071103 | DNA conformation change | 49/799 | 352/18862 | 1.99E-13 | 1.76E-11 | 49 |
| BP | GO:0090305 | nucleic acid phosphodiester bond hydrolysis | 45/799 | 305/18862 | 2.44E-13 | 2.11E-11 | 45 |
| BP | GO:1900034 | regulation of cellular response to heat | 22/799 | 79/18862 | 9.32E-13 | 7.85E-11 | 22 |
| BP | GO:0033044 | regulation of chromosome organization | 41/799 | 273/18862 | 1.59E-12 | 1.31E-10 | 41 |
| BP | GO:1901987 | regulation of cell cycle phase transition | 56/799 | 478/18862 | 5.18E-12 | 4.10E-10 | 56 |
| BP | GO:0031503 | protein-containing complex localization | 41/799 | 283/18862 | 5.23E-12 | 4.10E-10 | 41 |
| BP | GO:1901990 | regulation of mitotic cell cycle phase transition | 53/799 | 439/18862 | 6.18E-12 | 4.74E-10 | 53 |
| BP | GO:0034504 | protein localization to nucleus | 40/799 | 275/18862 | 8.39E-12 | 6.16E-10 | 40 |
| BP | GO:0006364 | rRNA processing | 36/799 | 228/18862 | 8.41E-12 | 6.16E-10 | 36 |
| BP | GO:0031123 | RNA 3'-end processing | 29/799 | 153/18862 | 9.42E-12 | 6.76E-10 | 29 |
| BP | GO:0031124 | mRNA 3'-end processing | 23/799 | 99/18862 | 1.74E-11 | 1.22E-09 | 23 |
| BP | GO:0006399 | tRNA metabolic process | 31/799 | 179/18862 | 2.11E-11 | 1.45E-09 | 31 |
| BP | GO:0098781 | ncRNA transcription | 24/799 | 109/18862 | 2.15E-11 | 1.45E-09 | 24 |
| BP | GO:0016072 | rRNA metabolic process | 36/799 | 238/18862 | 3.00E-11 | 1.98E-09 | 36 |
| BP | GO:0006260 | DNA replication | 39/799 | 280/18862 | 5.86E-11 | 3.80E-09 | 39 |
| BP | GO:0140053 | mitochondrial gene expression | 29/799 | 165/18862 | 6.47E-11 | 4.08E-09 | 29 |
| BP | GO:0071824 | protein-DNA complex subunit organization | 39/799 | 281/18862 | 6.54E-11 | 4.08E-09 | 39 |
| BP | GO:0060966 | regulation of gene silencing by RNA | 25/799 | 125/18862 | 7.60E-11 | 4.66E-09 | 25 |
| BP | GO:0051298 | centrosome duplication | 19/799 | 72/18862 | 9.36E-11 | 5.64E-09 | 19 |
| BP | GO:0072331 | signal transduction by p53 class mediator | 37/799 | 263/18862 | 1.37E-10 | 8.08E-09 | 37 |
| BP | GO:0006353 | DNA-templated transcription, termination | 19/799 | 75/18862 | 2.02E-10 | 1.18E-08 | 19 |
| BP | GO:0060964 | regulation of gene silencing by miRNA | 24/799 | 121/18862 | 2.18E-10 | 1.25E-08 | 24 |
| BP | GO:0098534 | centriole assembly | 15/799 | 45/18862 | 2.34E-10 | 1.32E-08 | 15 |
| BP | GO:0060968 | regulation of gene silencing | 26/799 | 144/18862 | 3.41E-10 | 1.89E-08 | 26 |
| BP | GO:0097711 | ciliary basal body-plasma membrane docking | 21/799 | 95/18862 | 3.56E-10 | 1.94E-08 | 21 |
| BP | GO:0060147 | regulation of posttranscriptional gene silencing | 24/799 | 124/18862 | 3.71E-10 | 1.98E-08 | 24 |
| BP | GO:0022618 | ribonucleoprotein complex assembly | 33/799 | 224/18862 | 4.06E-10 | 2.14E-08 | 33 |
| BP | GO:0006110 | regulation of glycolytic process | 19/799 | 79/18862 | 5.31E-10 | 2.76E-08 | 19 |
| BP | GO:0019083 | viral transcription | 29/799 | 180/18862 | 5.55E-10 | 2.83E-08 | 29 |
| BP | GO:0007099 | centriole replication | 14/799 | 41/18862 | 6.50E-10 | 3.27E-08 | 14 |
| BP | GO:0006401 | RNA catabolic process | 47/799 | 414/18862 | 7.98E-10 | 3.95E-08 | 47 |
| BP | GO:1903578 | regulation of ATP metabolic process | 23/799 | 119/18862 | 8.86E-10 | 4.33E-08 | 23 |
| BP | GO:0071826 | ribonucleoprotein complex subunit organization | 33/799 | 231/18862 | 9.14E-10 | 4.40E-08 | 33 |
| BP | GO:0034605 | cellular response to heat | 23/799 | 120/18862 | 1.05E-09 | 5.00E-08 | 23 |
| BP | GO:0019080 | viral gene expression | 30/799 | 198/18862 | 1.28E-09 | 6.01E-08 | 30 |
| BP | GO:0032259 | methylation | 42/799 | 359/18862 | 2.62E-09 | 1.18E-07 | 42 |
| BP | GO:0006378 | mRNA polyadenylation | 14/799 | 45/18862 | 2.63E-09 | 1.18E-07 | 14 |
| BP | GO:0043414 | macromolecule methylation | 38/799 | 305/18862 | 2.64E-09 | 1.18E-07 | 38 |
| BP | GO:0007051 | spindle organization | 28/799 | 182/18862 | 3.21E-09 | 1.42E-07 | 28 |
| BP | GO:0043631 | RNA polyadenylation | 14/799 | 46/18862 | 3.63E-09 | 1.59E-07 | 14 |
| BP | GO:0000723 | telomere maintenance | 26/799 | 161/18862 | 4.10E-09 | 1.77E-07 | 26 |
| BP | GO:0007059 | chromosome segregation | 40/799 | 337/18862 | 4.15E-09 | 1.77E-07 | 40 |
| BP | GO:0007098 | centrosome cycle | 23/799 | 129/18862 | 4.58E-09 | 1.93E-07 | 23 |
| BP | GO:0031023 | microtubule organizing center organization | 24/799 | 140/18862 | 4.79E-09 | 1.99E-07 | 24 |
| BP | GO:0000959 | mitochondrial RNA metabolic process | 14/799 | 47/18862 | 4.97E-09 | 2.04E-07 | 14 |
| BP | GO:0032200 | telomere organization | 27/799 | 174/18862 | 5.06E-09 | 2.05E-07 | 27 |
| BP | GO:0008033 | tRNA processing | 23/799 | 130/18862 | 5.35E-09 | 2.13E-07 | 23 |
| BP | GO:0051170 | import into nucleus | 26/799 | 163/18862 | 5.36E-09 | 2.13E-07 | 26 |
| BP | GO:0043470 | regulation of carbohydrate catabolic process | 19/799 | 90/18862 | 5.56E-09 | 2.18E-07 | 19 |
| BP | GO:0000070 | mitotic sister chromatid segregation | 26/799 | 164/18862 | 6.12E-09 | 2.37E-07 | 26 |
| BP | GO:0044786 | cell cycle DNA replication | 16/799 | 64/18862 | 6.73E-09 | 2.58E-07 | 16 |
| BP | GO:0006369 | termination of RNA polymerase II transcription | 12/799 | 35/18862 | 1.04E-08 | 3.95E-07 | 12 |
| BP | GO:0051225 | spindle assembly | 21/799 | 114/18862 | 1.17E-08 | 4.39E-07 | 21 |
| BP | GO:0140014 | mitotic nuclear division | 36/799 | 296/18862 | 1.31E-08 | 4.86E-07 | 36 |
| BP | GO:0051052 | regulation of DNA metabolic process | 40/799 | 353/18862 | 1.56E-08 | 5.71E-07 | 40 |
| BP | GO:0006417 | regulation of translation | 45/799 | 425/18862 | 1.60E-08 | 5.80E-07 | 45 |
| BP | GO:0090501 | RNA phosphodiester bond hydrolysis | 25/799 | 160/18862 | 1.62E-08 | 5.80E-07 | 25 |
| BP | GO:0032508 | DNA duplex unwinding | 20/799 | 107/18862 | 2.01E-08 | 7.14E-07 | 20 |
| BP | GO:0006999 | nuclear pore organization | 8/799 | 14/18862 | 2.40E-08 | 8.35E-07 | 8 |
| BP | GO:0000819 | sister chromatid segregation | 28/799 | 199/18862 | 2.40E-08 | 8.35E-07 | 28 |
| BP | GO:0006402 | mRNA catabolic process | 41/799 | 375/18862 | 2.86E-08 | 9.83E-07 | 41 |
| BP | GO:0070129 | regulation of mitochondrial translation | 10/799 | 25/18862 | 3.22E-08 | 1.10E-06 | 10 |
| BP | GO:0006606 | protein import into nucleus | 23/799 | 143/18862 | 3.47E-08 | 1.17E-06 | 23 |
| BP | GO:0006302 | double-strand break repair | 33/799 | 268/18862 | 3.92E-08 | 1.31E-06 | 33 |
| BP | GO:0032543 | mitochondrial translation | 22/799 | 134/18862 | 4.69E-08 | 1.55E-06 | 22 |
| BP | GO:0006261 | DNA-dependent DNA replication | 24/799 | 157/18862 | 4.81E-08 | 1.58E-06 | 24 |
| BP | GO:0009451 | RNA modification | 25/799 | 169/18862 | 4.95E-08 | 1.60E-06 | 25 |
| BP | GO:1902749 | regulation of cell cycle G2/M phase transition | 29/799 | 219/18862 | 5.31E-08 | 1.71E-06 | 29 |
| BP | GO:0071897 | DNA biosynthetic process | 27/799 | 194/18862 | 5.38E-08 | 1.71E-06 | 27 |
| BP | GO:0032392 | DNA geometric change | 20/799 | 114/18862 | 6.12E-08 | 1.93E-06 | 20 |
| BP | GO:0042795 | snRNA transcription by RNA polymerase II | 16/799 | 74/18862 | 6.23E-08 | 1.94E-06 | 16 |
| BP | GO:0043467 | regulation of generation of precursor metabolites and energy | 24/799 | 160/18862 | 6.97E-08 | 2.16E-06 | 24 |
| BP | GO:0009301 | snRNA transcription | 16/799 | 75/18862 | 7.61E-08 | 2.33E-06 | 16 |
| BP | GO:0061013 | regulation of mRNA catabolic process | 28/799 | 210/18862 | 7.76E-08 | 2.36E-06 | 28 |
| BP | GO:0009408 | response to heat | 24/799 | 161/18862 | 7.87E-08 | 2.37E-06 | 24 |
| BP | GO:0043487 | regulation of RNA stability | 27/799 | 198/18862 | 8.27E-08 | 2.47E-06 | 27 |
| BP | GO:0019058 | viral life cycle | 38/799 | 348/18862 | 9.51E-08 | 2.81E-06 | 38 |
| BP | GO:0034968 | histone lysine methylation | 19/799 | 107/18862 | 1.07E-07 | 3.13E-06 | 19 |
| BP | GO:0043488 | regulation of mRNA stability | 26/799 | 188/18862 | 1.08E-07 | 3.13E-06 | 26 |
| BP | GO:2001252 | positive regulation of chromosome organization | 24/799 | 164/18862 | 1.13E-07 | 3.24E-06 | 24 |
| BP | GO:0006323 | DNA packaging | 30/799 | 240/18862 | 1.16E-07 | 3.31E-06 | 30 |
| BP | GO:0007004 | telomere maintenance via telomerase | 15/799 | 68/18862 | 1.21E-07 | 3.43E-06 | 15 |
| BP | GO:1901796 | regulation of signal transduction by p53 class mediator | 25/799 | 177/18862 | 1.25E-07 | 3.47E-06 | 25 |
| BP | GO:0006289 | nucleotide-excision repair | 19/799 | 108/18862 | 1.25E-07 | 3.47E-06 | 19 |
| BP | GO:0016571 | histone methylation | 21/799 | 130/18862 | 1.26E-07 | 3.48E-06 | 21 |
| BP | GO:0010389 | regulation of G2/M transition of mitotic cell cycle | 27/799 | 203/18862 | 1.39E-07 | 3.81E-06 | 27 |
| BP | GO:1900542 | regulation of purine nucleotide metabolic process | 20/799 | 120/18862 | 1.48E-07 | 4.01E-06 | 20 |
| BP | GO:0062125 | regulation of mitochondrial gene expression | 10/799 | 29/18862 | 1.69E-07 | 4.56E-06 | 10 |
| BP | GO:0070646 | protein modification by small protein removal | 34/799 | 300/18862 | 1.86E-07 | 4.98E-06 | 34 |
| BP | GO:0006140 | regulation of nucleotide metabolic process | 20/799 | 122/18862 | 1.95E-07 | 5.18E-06 | 20 |
| BP | GO:0010833 | telomere maintenance via telomere lengthening | 16/799 | 80/18862 | 1.97E-07 | 5.18E-06 | 16 |
| BP | GO:0043484 | regulation of RNA splicing | 22/799 | 145/18862 | 1.98E-07 | 5.18E-06 | 22 |
| BP | GO:0010948 | negative regulation of cell cycle process | 38/799 | 359/18862 | 2.13E-07 | 5.52E-06 | 38 |
| BP | GO:0018022 | peptidyl-lysine methylation | 20/799 | 123/18862 | 2.24E-07 | 5.76E-06 | 20 |
| BP | GO:0006333 | chromatin assembly or disassembly | 28/799 | 221/18862 | 2.30E-07 | 5.87E-06 | 28 |
| BP | GO:0000075 | cell cycle checkpoint | 27/799 | 209/18862 | 2.53E-07 | 6.35E-06 | 27 |
| BP | GO:0006338 | chromatin remodeling | 27/799 | 209/18862 | 2.53E-07 | 6.35E-06 | 27 |
| BP | GO:0044839 | cell cycle G2/M phase transition | 32/799 | 276/18862 | 2.54E-07 | 6.35E-06 | 32 |
| BP | GO:0006352 | DNA-templated transcription, initiation | 30/799 | 249/18862 | 2.61E-07 | 6.47E-06 | 30 |
| BP | GO:0140056 | organelle localization by membrane tethering | 24/799 | 172/18862 | 2.79E-07 | 6.87E-06 | 24 |
| BP | GO:1902275 | regulation of chromatin organization | 25/799 | 185/18862 | 2.96E-07 | 7.22E-06 | 25 |
| BP | GO:0006096 | glycolytic process | 19/799 | 114/18862 | 3.01E-07 | 7.30E-06 | 19 |
| BP | GO:0098789 | pre-mRNA cleavage required for polyadenylation | 7/799 | 13/18862 | 3.27E-07 | 7.88E-06 | 7 |
| BP | GO:0045787 | positive regulation of cell cycle | 40/799 | 395/18862 | 3.31E-07 | 7.91E-06 | 40 |
| BP | GO:0006757 | ATP generation from ADP | 19/799 | 115/18862 | 3.47E-07 | 8.23E-06 | 19 |
| BP | GO:0006278 | RNA-dependent DNA biosynthetic process | 15/799 | 75/18862 | 4.73E-07 | 1.11E-05 | 15 |
| BP | GO:0042274 | ribosomal small subunit biogenesis | 15/799 | 75/18862 | 4.73E-07 | 1.11E-05 | 15 |
| BP | GO:0034248 | regulation of cellular amide metabolic process | 46/799 | 494/18862 | 5.02E-07 | 1.17E-05 | 46 |
| BP | GO:0098813 | nuclear chromosome segregation | 31/799 | 273/18862 | 6.17E-07 | 1.42E-05 | 31 |
| BP | GO:0098787 | mRNA cleavage involved in mRNA processing | 7/799 | 14/18862 | 6.30E-07 | 1.45E-05 | 7 |
| BP | GO:0000288 | nuclear-transcribed mRNA catabolic process, deadenylation-dependent decay | 15/799 | 77/18862 | 6.76E-07 | 1.54E-05 | 15 |
| BP | GO:0090068 | positive regulation of cell cycle process | 33/799 | 303/18862 | 7.02E-07 | 1.59E-05 | 33 |
| BP | GO:0006165 | nucleoside diphosphate phosphorylation | 20/799 | 132/18862 | 7.22E-07 | 1.60E-05 | 20 |
| BP | GO:0031570 | DNA integrity checkpoint | 22/799 | 156/18862 | 7.23E-07 | 1.60E-05 | 22 |
| BP | GO:0022406 | membrane docking | 24/799 | 181/18862 | 7.23E-07 | 1.60E-05 | 24 |
| BP | GO:0017038 | protein import | 25/799 | 194/18862 | 7.34E-07 | 1.62E-05 | 25 |
| BP | GO:1903312 | negative regulation of mRNA metabolic process | 16/799 | 88/18862 | 7.70E-07 | 1.69E-05 | 16 |
| BP | GO:0046939 | nucleotide phosphorylation | 20/799 | 133/18862 | 8.17E-07 | 1.78E-05 | 20 |
| BP | GO:0030490 | maturation of SSU-rRNA | 12/799 | 50/18862 | 8.45E-07 | 1.82E-05 | 12 |
| BP | GO:0033260 | nuclear DNA replication | 13/799 | 59/18862 | 8.53E-07 | 1.82E-05 | 13 |
| BP | GO:0006479 | protein methylation | 23/799 | 170/18862 | 8.58E-07 | 1.82E-05 | 23 |
| BP | GO:0008213 | protein alkylation | 23/799 | 170/18862 | 8.58E-07 | 1.82E-05 | 23 |
| BP | GO:0046031 | ADP metabolic process | 19/799 | 122/18862 | 8.87E-07 | 1.87E-05 | 19 |
| BP | GO:0043044 | ATP-dependent chromatin remodeling | 16/799 | 89/18862 | 9.03E-07 | 1.89E-05 | 16 |
| BP | GO:0007093 | mitotic cell cycle checkpoint | 22/799 | 159/18862 | 1.01E-06 | 2.09E-05 | 22 |
| BP | GO:1904356 | regulation of telomere maintenance via telomere lengthening | 13/799 | 60/18862 | 1.05E-06 | 2.17E-05 | 13 |
| BP | GO:1904874 | positive regulation of telomerase RNA localization to Cajal body | 7/799 | 15/18862 | 1.14E-06 | 2.34E-05 | 7 |
| BP | GO:0046599 | regulation of centriole replication | 8/799 | 21/18862 | 1.25E-06 | 2.55E-05 | 8 |
| BP | GO:0032210 | regulation of telomere maintenance via telomerase | 12/799 | 52/18862 | 1.33E-06 | 2.70E-05 | 12 |
| BP | GO:0065004 | protein-DNA complex assembly | 28/799 | 241/18862 | 1.36E-06 | 2.74E-05 | 28 |
| BP | GO:0046822 | regulation of nucleocytoplasmic transport | 17/799 | 103/18862 | 1.45E-06 | 2.90E-05 | 17 |
| BP | GO:0006310 | DNA recombination | 32/799 | 299/18862 | 1.51E-06 | 3.02E-05 | 32 |
| BP | GO:0010824 | regulation of centrosome duplication | 11/799 | 44/18862 | 1.57E-06 | 3.11E-05 | 11 |
| BP | GO:0000086 | G2/M transition of mitotic cell cycle | 29/799 | 258/18862 | 1.74E-06 | 3.43E-05 | 29 |
| BP | GO:0006379 | mRNA cleavage | 8/799 | 22/18862 | 1.89E-06 | 3.68E-05 | 8 |
| BP | GO:0048285 | organelle fission | 44/799 | 486/18862 | 1.89E-06 | 3.68E-05 | 44 |
| BP | GO:0016573 | histone acetylation | 21/799 | 153/18862 | 2.01E-06 | 3.90E-05 | 21 |
| BP | GO:0000082 | G1/S transition of mitotic cell cycle | 30/799 | 275/18862 | 2.16E-06 | 4.16E-05 | 30 |
| BP | GO:0018394 | peptidyl-lysine acetylation | 22/799 | 168/18862 | 2.57E-06 | 4.91E-05 | 22 |
| BP | GO:0034728 | nucleosome organization | 23/799 | 183/18862 | 3.12E-06 | 5.94E-05 | 23 |
| BP | GO:0018393 | internal peptidyl-lysine acetylation | 21/799 | 158/18862 | 3.40E-06 | 6.43E-05 | 21 |
| BP | GO:0006284 | base-excision repair | 10/799 | 39/18862 | 3.66E-06 | 6.88E-05 | 10 |
| BP | GO:0046605 | regulation of centrosome cycle | 11/799 | 48/18862 | 3.95E-06 | 7.40E-05 | 11 |
| BP | GO:0044843 | cell cycle G1/S phase transition | 31/799 | 298/18862 | 3.98E-06 | 7.41E-05 | 31 |
| BP | GO:0006475 | internal protein amino acid acetylation | 21/799 | 160/18862 | 4.16E-06 | 7.70E-05 | 21 |
| BP | GO:0009135 | purine nucleoside diphosphate metabolic process | 19/799 | 135/18862 | 4.20E-06 | 7.70E-05 | 19 |
| BP | GO:0009179 | purine ribonucleoside diphosphate metabolic process | 19/799 | 135/18862 | 4.20E-06 | 7.70E-05 | 19 |
| BP | GO:0006473 | protein acetylation | 24/799 | 200/18862 | 4.34E-06 | 7.91E-05 | 24 |
| BP | GO:0048024 | regulation of mRNA splicing, via spliceosome | 16/799 | 100/18862 | 4.46E-06 | 8.06E-05 | 16 |
| BP | GO:0000077 | DNA damage checkpoint | 20/799 | 148/18862 | 4.47E-06 | 8.06E-05 | 20 |
| BP | GO:0006400 | tRNA modification | 15/799 | 89/18862 | 4.56E-06 | 8.17E-05 | 15 |
| BP | GO:1904872 | regulation of telomerase RNA localization to Cajal body | 7/799 | 18/18862 | 5.04E-06 | 8.98E-05 | 7 |
| BP | GO:0032204 | regulation of telomere maintenance | 14/799 | 79/18862 | 5.14E-06 | 9.12E-05 | 14 |
| BP | GO:0044773 | mitotic DNA damage checkpoint | 16/799 | 102/18862 | 5.80E-06 | 0.000102434 | 16 |
| BP | GO:0009185 | ribonucleoside diphosphate metabolic process | 19/799 | 138/18862 | 5.84E-06 | 0.00010253 | 19 |
| BP | GO:0032212 | positive regulation of telomere maintenance via telomerase | 9/799 | 33/18862 | 6.47E-06 | 0.000113035 | 9 |
| BP | GO:0006109 | regulation of carbohydrate metabolic process | 24/799 | 206/18862 | 7.25E-06 | 0.000126 | 24 |
| BP | GO:0046831 | regulation of RNA export from nucleus | 6/799 | 13/18862 | 7.54E-06 | 0.000129485 | 6 |
| BP | GO:0006271 | DNA strand elongation involved in DNA replication | 7/799 | 19/18862 | 7.68E-06 | 0.000129485 | 7 |
| BP | GO:0090670 | RNA localization to Cajal body | 7/799 | 19/18862 | 7.68E-06 | 0.000129485 | 7 |
| BP | GO:0090671 | telomerase RNA localization to Cajal body | 7/799 | 19/18862 | 7.68E-06 | 0.000129485 | 7 |
| BP | GO:0090672 | telomerase RNA localization | 7/799 | 19/18862 | 7.68E-06 | 0.000129485 | 7 |
| BP | GO:0090685 | RNA localization to nucleus | 7/799 | 19/18862 | 7.68E-06 | 0.000129485 | 7 |
| BP | GO:0031056 | regulation of histone modification | 19/799 | 141/18862 | 8.03E-06 | 0.000134659 | 19 |
| BP | GO:0000280 | nuclear division | 39/799 | 436/18862 | 9.57E-06 | 0.000159712 | 39 |
| BP | GO:0044774 | mitotic DNA integrity checkpoint | 16/799 | 106/18862 | 9.64E-06 | 0.00016002 | 16 |
| BP | GO:0009132 | nucleoside diphosphate metabolic process | 20/799 | 156/18862 | 1.00E-05 | 0.000165424 | 20 |
| BP | GO:0062012 | regulation of small molecule metabolic process | 39/799 | 437/18862 | 1.01E-05 | 0.000165967 | 39 |
| BP | GO:0006283 | transcription-coupled nucleotide-excision repair | 13/799 | 73/18862 | 1.05E-05 | 0.000172118 | 13 |
| BP | GO:0016052 | carbohydrate catabolic process | 23/799 | 197/18862 | 1.08E-05 | 0.00017533 | 23 |
| BP | GO:0016579 | protein deubiquitination | 29/799 | 283/18862 | 1.08E-05 | 0.00017533 | 29 |
| BP | GO:2000278 | regulation of DNA biosynthetic process | 16/799 | 107/18862 | 1.09E-05 | 0.000175687 | 16 |
| BP | GO:0000291 | nuclear-transcribed mRNA catabolic process, exonucleolytic | 9/799 | 35/18862 | 1.10E-05 | 0.000176241 | 9 |
| BP | GO:0046931 | pore complex assembly | 7/799 | 20/18862 | 1.14E-05 | 0.00018193 | 7 |
| BP | GO:0051054 | positive regulation of DNA metabolic process | 23/799 | 198/18862 | 1.17E-05 | 0.000186677 | 23 |
| BP | GO:0009266 | response to temperature stimulus | 25/799 | 228/18862 | 1.37E-05 | 0.000216554 | 25 |
| BP | GO:1904358 | positive regulation of telomere maintenance via telomere lengthening | 9/799 | 36/18862 | 1.41E-05 | 0.000221986 | 9 |
| BP | GO:0031440 | regulation of mRNA 3'-end processing | 8/799 | 28/18862 | 1.46E-05 | 0.000228105 | 8 |
| BP | GO:0051569 | regulation of histone H3-K4 methylation | 8/799 | 28/18862 | 1.46E-05 | 0.000228105 | 8 |
| BP | GO:0033962 | P-body assembly | 7/799 | 21/18862 | 1.65E-05 | 0.000255671 | 7 |
| BP | GO:0006997 | nucleus organization | 17/799 | 124/18862 | 1.89E-05 | 0.000292597 | 17 |
| BP | GO:0050686 | negative regulation of mRNA processing | 8/799 | 29/18862 | 1.94E-05 | 0.000299209 | 8 |
| BP | GO:0006090 | pyruvate metabolic process | 19/799 | 150/18862 | 1.97E-05 | 0.000301991 | 19 |
| BP | GO:0006388 | tRNA splicing, via endonucleolytic cleavage and ligation | 6/799 | 15/18862 | 2.04E-05 | 0.000311583 | 6 |
| BP | GO:0000724 | double-strand break repair via homologous recombination | 18/799 | 138/18862 | 2.17E-05 | 0.000329363 | 18 |
| BP | GO:0006360 | transcription by RNA polymerase I | 12/799 | 68/18862 | 2.52E-05 | 0.000380647 | 12 |
| BP | GO:0000725 | recombinational repair | 18/799 | 140/18862 | 2.64E-05 | 0.000397555 | 18 |
| BP | GO:0000491 | small nucleolar ribonucleoprotein complex assembly | 5/799 | 10/18862 | 2.84E-05 | 0.000425687 | 5 |
| BP | GO:0000394 | RNA splicing, via endonucleolytic cleavage and ligation | 6/799 | 16/18862 | 3.15E-05 | 0.000465926 | 6 |
| BP | GO:0032239 | regulation of nucleobase-containing compound transport | 6/799 | 16/18862 | 3.15E-05 | 0.000465926 | 6 |
| BP | GO:0070131 | positive regulation of mitochondrial translation | 6/799 | 16/18862 | 3.15E-05 | 0.000465926 | 6 |
| BP | GO:1902402 | signal transduction involved in mitotic DNA damage checkpoint | 11/799 | 59/18862 | 3.20E-05 | 0.00046903 | 11 |
| BP | GO:1902403 | signal transduction involved in mitotic DNA integrity checkpoint | 11/799 | 59/18862 | 3.20E-05 | 0.00046903 | 11 |
| BP | GO:1902850 | microtubule cytoskeleton organization involved in mitosis | 18/799 | 143/18862 | 3.52E-05 | 0.000514147 | 18 |
| BP | GO:0043543 | protein acylation | 25/799 | 242/18862 | 3.76E-05 | 0.000546826 | 25 |
| BP | GO:0007052 | mitotic spindle organization | 16/799 | 118/18862 | 3.79E-05 | 0.000548733 | 16 |
| BP | GO:0006354 | DNA-templated transcription, elongation | 16/799 | 119/18862 | 4.21E-05 | 0.000606902 | 16 |
| BP | GO:0031060 | regulation of histone methylation | 11/799 | 61/18862 | 4.43E-05 | 0.000632189 | 11 |
| BP | GO:0072413 | signal transduction involved in mitotic cell cycle checkpoint | 11/799 | 61/18862 | 4.43E-05 | 0.000632189 | 11 |
| BP | GO:0051304 | chromosome separation | 14/799 | 95/18862 | 4.51E-05 | 0.000641719 | 14 |
| BP | GO:0042273 | ribosomal large subunit biogenesis | 12/799 | 72/18862 | 4.55E-05 | 0.000644352 | 12 |
| BP | GO:0032206 | positive regulation of telomere maintenance | 10/799 | 51/18862 | 4.64E-05 | 0.000653804 | 10 |
| BP | GO:1900363 | regulation of mRNA polyadenylation | 6/799 | 17/18862 | 4.70E-05 | 0.000659592 | 6 |
| BP | GO:0045727 | positive regulation of translation | 17/799 | 133/18862 | 4.74E-05 | 0.000662227 | 17 |
| BP | GO:0001510 | RNA methylation | 13/799 | 84/18862 | 4.97E-05 | 0.000692193 | 13 |
| BP | GO:0031571 | mitotic G1 DNA damage checkpoint | 11/799 | 62/18862 | 5.18E-05 | 0.00071768 | 11 |
| BP | GO:0000380 | alternative mRNA splicing, via spliceosome | 12/799 | 73/18862 | 5.24E-05 | 0.00071768 | 12 |
| BP | GO:0072401 | signal transduction involved in DNA integrity checkpoint | 12/799 | 73/18862 | 5.24E-05 | 0.00071768 | 12 |
| BP | GO:0072422 | signal transduction involved in DNA damage checkpoint | 12/799 | 73/18862 | 5.24E-05 | 0.00071768 | 12 |
| BP | GO:0009303 | rRNA transcription | 8/799 | 33/18862 | 5.41E-05 | 0.000729483 | 8 |
| BP | GO:0043928 | exonucleolytic catabolism of deadenylated mRNA | 8/799 | 33/18862 | 5.41E-05 | 0.000729483 | 8 |
| BP | GO:0051973 | positive regulation of telomerase activity | 8/799 | 33/18862 | 5.41E-05 | 0.000729483 | 8 |
| BP | GO:0071539 | protein localization to centrosome | 8/799 | 33/18862 | 5.41E-05 | 0.000729483 | 8 |
| BP | GO:0007088 | regulation of mitotic nuclear division | 15/799 | 109/18862 | 5.53E-05 | 0.000742109 | 15 |
| BP | GO:0000729 | DNA double-strand break processing | 7/799 | 25/18862 | 5.87E-05 | 0.000784629 | 7 |
| BP | GO:0044783 | G1 DNA damage checkpoint | 11/799 | 63/18862 | 6.04E-05 | 0.000800979 | 11 |
| BP | GO:0044819 | mitotic G1/S transition checkpoint | 11/799 | 63/18862 | 6.04E-05 | 0.000800979 | 11 |
| BP | GO:0090503 | RNA phosphodiester bond hydrolysis, exonucleolytic | 9/799 | 43/18862 | 6.47E-05 | 0.000855336 | 9 |
| BP | GO:0051568 | histone H3-K4 methylation | 10/799 | 53/18862 | 6.55E-05 | 0.000862848 | 10 |
| BP | GO:0006337 | nucleosome disassembly | 6/799 | 18/18862 | 6.79E-05 | 0.000887462 | 6 |
| BP | GO:0051571 | positive regulation of histone H3-K4 methylation | 6/799 | 18/18862 | 6.79E-05 | 0.000887462 | 6 |
| BP | GO:0000956 | nuclear-transcribed mRNA catabolic process | 22/799 | 208/18862 | 7.62E-05 | 0.000991222 | 22 |
| BP | GO:0022616 | DNA strand elongation | 7/799 | 26/18862 | 7.74E-05 | 0.001002826 | 7 |
| BP | GO:0072395 | signal transduction involved in cell cycle checkpoint | 12/799 | 76/18862 | 7.87E-05 | 0.001016169 | 12 |
| BP | GO:1905269 | positive regulation of chromatin organization | 14/799 | 100/18862 | 8.02E-05 | 0.001032202 | 14 |
| BP | GO:2000573 | positive regulation of DNA biosynthetic process | 11/799 | 65/18862 | 8.12E-05 | 0.001041076 | 11 |
| BP | GO:0010826 | negative regulation of centrosome duplication | 5/799 | 12/18862 | 8.32E-05 | 0.001054064 | 5 |
| BP | GO:0046606 | negative regulation of centrosome cycle | 5/799 | 12/18862 | 8.32E-05 | 0.001054064 | 5 |
| BP | GO:1904816 | positive regulation of protein localization to chromosome, telomeric region | 5/799 | 12/18862 | 8.32E-05 | 0.001054064 | 5 |
| BP | GO:1905508 | protein localization to microtubule organizing center | 8/799 | 35/18862 | 8.51E-05 | 0.001074116 | 8 |
| BP | GO:0031497 | chromatin assembly | 21/799 | 196/18862 | 9.15E-05 | 0.001150163 | 21 |
| BP | GO:0051983 | regulation of chromosome segregation | 13/799 | 89/18862 | 9.19E-05 | 0.001151748 | 13 |
| BP | GO:0051306 | mitotic sister chromatid separation | 11/799 | 66/18862 | 9.38E-05 | 0.001170964 | 11 |
| BP | GO:0032886 | regulation of microtubule-based process | 23/799 | 227/18862 | 0.000102109 | 0.001269963 | 23 |
| BP | GO:0034508 | centromere complex assembly | 10/799 | 56/18862 | 0.000106708 | 0.001322287 | 10 |
| BP | GO:0045930 | negative regulation of mitotic cell cycle | 29/799 | 321/18862 | 0.000109116 | 0.001347172 | 29 |
| BP | GO:1901992 | positive regulation of mitotic cell cycle phase transition | 13/799 | 91/18862 | 0.000115971 | 0.001426582 | 13 |
| BP | GO:0000381 | regulation of alternative mRNA splicing, via spliceosome | 10/799 | 57/18862 | 0.000124562 | 0.00151567 | 10 |
| BP | GO:0072431 | signal transduction involved in mitotic G1 DNA damage checkpoint | 10/799 | 57/18862 | 0.000124562 | 0.00151567 | 10 |
| BP | GO:1902400 | intracellular signal transduction involved in G1 DNA damage checkpoint | 10/799 | 57/18862 | 0.000124562 | 0.00151567 | 10 |
| BP | GO:0000462 | maturation of SSU-rRNA from tricistronic rRNA transcript (SSU-rRNA, 5.8S rRNA, LSU-rRNA) | 8/799 | 37/18862 | 0.000129493 | 0.001569998 | 8 |
| BP | GO:0032042 | mitochondrial DNA metabolic process | 5/799 | 13/18862 | 0.000130468 | 0.00157615 | 5 |
| BP | GO:0031498 | chromatin disassembly | 6/799 | 20/18862 | 0.000131888 | 0.001581967 | 6 |
| BP | GO:0032986 | protein-DNA complex disassembly | 6/799 | 20/18862 | 0.000131888 | 0.001581967 | 6 |
| BP | GO:0051972 | regulation of telomerase activity | 9/799 | 47/18862 | 0.000134373 | 0.001606053 | 9 |
| BP | GO:1902115 | regulation of organelle assembly | 20/799 | 187/18862 | 0.000136574 | 0.001626593 | 20 |
| BP | GO:1902369 | negative regulation of RNA catabolic process | 11/799 | 70/18862 | 0.000162034 | 0.001923031 | 11 |
| BP | GO:1901989 | positive regulation of cell cycle phase transition | 14/799 | 107/18862 | 0.000168065 | 0.001981765 | 14 |
| BP | GO:0046034 | ATP metabolic process | 28/799 | 313/18862 | 0.000168159 | 0.001981765 | 28 |
| BP | GO:0000002 | mitochondrial genome maintenance | 6/799 | 21/18862 | 0.000178059 | 0.002091127 | 6 |
| BP | GO:1905818 | regulation of chromosome separation | 11/799 | 71/18862 | 0.000184515 | 0.002159415 | 11 |
| BP | GO:0090224 | regulation of spindle organization | 8/799 | 39/18862 | 0.000191461 | 0.002232955 | 8 |
| BP | GO:0043966 | histone H3 acetylation | 10/799 | 60/18862 | 0.000193949 | 0.002253388 | 10 |
| BP | GO:0000054 | ribosomal subunit export from nucleus | 5/799 | 14/18862 | 0.000195887 | 0.002253388 | 5 |
| BP | GO:0033750 | ribosome localization | 5/799 | 14/18862 | 0.000195887 | 0.002253388 | 5 |
| BP | GO:1904814 | regulation of protein localization to chromosome, telomeric region | 5/799 | 14/18862 | 0.000195887 | 0.002253388 | 5 |
| BP | GO:1903313 | positive regulation of mRNA metabolic process | 12/799 | 84/18862 | 0.000210361 | 0.002411655 | 12 |
| BP | GO:0007091 | metaphase/anaphase transition of mitotic cell cycle | 10/799 | 61/18862 | 0.000223299 | 0.002551308 | 10 |
| BP | GO:0051383 | kinetochore organization | 6/799 | 22/18862 | 0.000236108 | 0.002688544 | 6 |
| BP | GO:0045070 | positive regulation of viral genome replication | 7/799 | 31/18862 | 0.000257055 | 0.002907414 | 7 |
| BP | GO:1904837 | beta-catenin-TCF complex assembly | 7/799 | 31/18862 | 0.000257055 | 0.002907414 | 7 |
| BP | GO:0070317 | negative regulation of G0 to G1 transition | 8/799 | 41/18862 | 0.000275908 | 0.003110223 | 8 |
| BP | GO:0007076 | mitotic chromosome condensation | 5/799 | 15/18862 | 0.000283616 | 0.003175861 | 5 |
| BP | GO:0071428 | rRNA-containing ribonucleoprotein complex export from nucleus | 5/799 | 15/18862 | 0.000283616 | 0.003175861 | 5 |
| BP | GO:0006297 | nucleotide-excision repair, DNA gap filling | 6/799 | 23/18862 | 0.000308068 | 0.003426906 | 6 |
| BP | GO:0045943 | positive regulation of transcription by RNA polymerase I | 6/799 | 23/18862 | 0.000308068 | 0.003426906 | 6 |
| BP | GO:0010965 | regulation of mitotic sister chromatid separation | 10/799 | 64/18862 | 0.000334568 | 0.003697281 | 10 |
| BP | GO:0044784 | metaphase/anaphase transition of cell cycle | 10/799 | 64/18862 | 0.000334568 | 0.003697281 | 10 |
| BP | GO:1900180 | regulation of protein localization to nucleus | 15/799 | 130/18862 | 0.000404572 | 0.004456273 | 15 |
| BP | GO:1901991 | negative regulation of mitotic cell cycle phase transition | 23/799 | 250/18862 | 0.000420039 | 0.004611571 | 23 |
| BP | GO:2001251 | negative regulation of chromosome organization | 12/799 | 91/18862 | 0.00044839 | 0.004906849 | 12 |
| BP | GO:0040029 | regulation of gene expression, epigenetic | 20/799 | 205/18862 | 0.000463195 | 0.005048527 | 20 |
| BP | GO:0019693 | ribose phosphate metabolic process | 34/799 | 435/18862 | 0.000464332 | 0.005048527 | 34 |
| BP | GO:0045931 | positive regulation of mitotic cell cycle | 14/799 | 118/18862 | 0.000469825 | 0.005091057 | 14 |
| BP | GO:0009411 | response to UV | 16/799 | 146/18862 | 0.000471265 | 0.005091057 | 16 |
| BP | GO:0001672 | regulation of chromatin assembly or disassembly | 6/799 | 25/18862 | 0.000502721 | 0.005396286 | 6 |
| BP | GO:0090169 | regulation of spindle assembly | 6/799 | 25/18862 | 0.000502721 | 0.005396286 | 6 |
| BP | GO:0006977 | DNA damage response, signal transduction by p53 class mediator resulting in cell cycle arrest | 9/799 | 56/18862 | 0.000531731 | 0.005689568 | 9 |
| BP | GO:0042790 | nucleolar large rRNA transcription by RNA polymerase I | 5/799 | 17/18862 | 0.000544555 | 0.005777781 | 5 |
| BP | GO:2000104 | negative regulation of DNA-dependent DNA replication | 5/799 | 17/18862 | 0.000544555 | 0.005777781 | 5 |
| BP | GO:1901838 | positive regulation of transcription of nucleolar large rRNA by RNA polymerase I | 4/799 | 10/18862 | 0.000546832 | 0.005777781 | 4 |
| BP | GO:1904851 | positive regulation of establishment of protein localization to telomere | 4/799 | 10/18862 | 0.000546832 | 0.005777781 | 4 |
| BP | GO:0034250 | positive regulation of cellular amide metabolic process | 17/799 | 163/18862 | 0.000566044 | 0.005956294 | 17 |
| BP | GO:0000289 | nuclear-transcribed mRNA poly(A) tail shortening | 7/799 | 35/18862 | 0.000567262 | 0.005956294 | 7 |
| BP | GO:0001890 | placenta development | 15/799 | 135/18862 | 0.000605563 | 0.006310893 | 15 |
| BP | GO:0051783 | regulation of nuclear division | 15/799 | 135/18862 | 0.000605563 | 0.006310893 | 15 |
| BP | GO:0006304 | DNA modification | 14/799 | 121/18862 | 0.00060665 | 0.006310893 | 14 |
| BP | GO:0030261 | chromosome condensation | 8/799 | 46/18862 | 0.000625166 | 0.006463619 | 8 |
| BP | GO:0070316 | regulation of G0 to G1 transition | 8/799 | 46/18862 | 0.000625166 | 0.006463619 | 8 |
| BP | GO:0009259 | ribonucleotide metabolic process | 33/799 | 425/18862 | 0.000627106 | 0.006463843 | 33 |
| BP | GO:0070911 | global genome nucleotide-excision repair | 6/799 | 26/18862 | 0.00063033 | 0.006477266 | 6 |
| BP | GO:0071158 | positive regulation of cell cycle arrest | 11/799 | 82/18862 | 0.000661887 | 0.006780876 | 11 |
| BP | GO:0045023 | G0 to G1 transition | 8/799 | 47/18862 | 0.000725842 | 0.007368884 | 8 |
| BP | GO:0045839 | negative regulation of mitotic nuclear division | 8/799 | 47/18862 | 0.000725842 | 0.007368884 | 8 |
| BP | GO:0090329 | regulation of DNA-dependent DNA replication | 8/799 | 47/18862 | 0.000725842 | 0.007368884 | 8 |
| BP | GO:0033045 | regulation of sister chromatid segregation | 10/799 | 71/18862 | 0.000781549 | 0.0078646 | 10 |
| BP | GO:0010971 | positive regulation of G2/M transition of mitotic cell cycle | 6/799 | 27/18862 | 0.00078167 | 0.0078646 | 6 |
| BP | GO:0032201 | telomere maintenance via semi-conservative replication | 6/799 | 27/18862 | 0.00078167 | 0.0078646 | 6 |
| BP | GO:0030071 | regulation of mitotic metaphase/anaphase transition | 9/799 | 59/18862 | 0.000787697 | 0.007878199 | 9 |
| BP | GO:1902373 | negative regulation of mRNA catabolic process | 9/799 | 59/18862 | 0.000787697 | 0.007878199 | 9 |
| BP | GO:0006296 | nucleotide-excision repair, DNA incision, 5'-to lesion | 7/799 | 37/18862 | 0.000806913 | 0.008046514 | 7 |
| BP | GO:0070203 | regulation of establishment of protein localization to telomere | 4/799 | 11/18862 | 0.000830577 | 0.008209624 | 4 |
| BP | GO:1904869 | regulation of protein localization to Cajal body | 4/799 | 11/18862 | 0.000830577 | 0.008209624 | 4 |
| BP | GO:1904871 | positive regulation of protein localization to Cajal body | 4/799 | 11/18862 | 0.000830577 | 0.008209624 | 4 |
| BP | GO:0043039 | tRNA aminoacylation | 8/799 | 48/18862 | 0.000839163 | 0.008270233 | 8 |
| BP | GO:0043489 | RNA stabilization | 9/799 | 60/18862 | 0.000892476 | 0.00877001 | 9 |
| BP | GO:0006298 | mismatch repair | 7/799 | 38/18862 | 0.000953443 | 0.0093148 | 7 |
| BP | GO:0006361 | transcription initiation from RNA polymerase I promoter | 7/799 | 38/18862 | 0.000953443 | 0.0093148 | 7 |
| BP | GO:0044728 | DNA methylation or demethylation | 12/799 | 99/18862 | 0.000965638 | 0.009358566 | 12 |
| BP | GO:0043038 | amino acid activation | 8/799 | 49/18862 | 0.000966253 | 0.009358566 | 8 |
| BP | GO:0045005 | DNA-dependent DNA replication maintenance of fidelity | 8/799 | 49/18862 | 0.000966253 | 0.009358566 | 8 |
| BP | GO:0046824 | positive regulation of nucleocytoplasmic transport | 9/799 | 61/18862 | 0.001008295 | 0.009737778 | 9 |
| BP | GO:0006367 | transcription initiation from RNA polymerase II promoter | 18/799 | 187/18862 | 0.001020279 | 0.009825368 | 18 |
| BP | GO:1901988 | negative regulation of cell cycle phase transition | 23/799 | 268/18862 | 0.001093674 | 0.01050216 | 23 |
| BP | GO:0033683 | nucleotide-excision repair, DNA incision | 7/799 | 39/18862 | 0.001120127 | 0.010725618 | 7 |
| BP | GO:1902099 | regulation of metaphase/anaphase transition of cell cycle | 9/799 | 62/18862 | 0.001135995 | 0.010846744 | 9 |
| BP | GO:0006294 | nucleotide-excision repair, preincision complex assembly | 6/799 | 29/18862 | 0.001167061 | 0.011111891 | 6 |
| BP | GO:2001020 | regulation of response to DNA damage stimulus | 20/799 | 221/18862 | 0.001189267 | 0.011255797 | 20 |
| BP | GO:0014029 | neural crest formation | 4/799 | 12/18862 | 0.001204272 | 0.011255797 | 4 |
| BP | GO:0070202 | regulation of establishment of protein localization to chromosome | 4/799 | 12/18862 | 0.001204272 | 0.011255797 | 4 |
| BP | GO:1903405 | protein localization to nuclear body | 4/799 | 12/18862 | 0.001204272 | 0.011255797 | 4 |
| BP | GO:1904867 | protein localization to Cajal body | 4/799 | 12/18862 | 0.001204272 | 0.011255797 | 4 |
| BP | GO:0006305 | DNA alkylation | 10/799 | 75/18862 | 0.001205551 | 0.011255797 | 10 |
| BP | GO:0006306 | DNA methylation | 10/799 | 75/18862 | 0.001205551 | 0.011255797 | 10 |
| BP | GO:0072521 | purine-containing compound metabolic process | 34/799 | 460/18862 | 0.001226551 | 0.011335584 | 34 |
| BP | GO:0006450 | regulation of translational fidelity | 5/799 | 20/18862 | 0.00122755 | 0.011335584 | 5 |
| BP | GO:0010847 | regulation of chromatin assembly | 5/799 | 20/18862 | 0.00122755 | 0.011335584 | 5 |
| BP | GO:0048025 | negative regulation of mRNA splicing, via spliceosome | 5/799 | 20/18862 | 0.00122755 | 0.011335584 | 5 |
| BP | GO:0006282 | regulation of DNA repair | 14/799 | 130/18862 | 0.001235595 | 0.0113787 | 14 |
| BP | GO:0031330 | negative regulation of cellular catabolic process | 22/799 | 255/18862 | 0.001297722 | 0.011918273 | 22 |
| BP | GO:0006270 | DNA replication initiation | 7/799 | 40/18862 | 0.001308828 | 0.011955122 | 7 |
| BP | GO:0030488 | tRNA methylation | 7/799 | 40/18862 | 0.001308828 | 0.011955122 | 7 |
| BP | GO:0042770 | signal transduction in response to DNA damage | 14/799 | 131/18862 | 0.001330866 | 0.012123565 | 14 |
| BP | GO:0007020 | microtubule nucleation | 6/799 | 30/18862 | 0.001407203 | 0.012715855 | 6 |
| BP | GO:0070198 | protein localization to chromosome, telomeric region | 6/799 | 30/18862 | 0.001407203 | 0.012715855 | 6 |
| BP | GO:1902751 | positive regulation of cell cycle G2/M phase transition | 6/799 | 30/18862 | 0.001407203 | 0.012715855 | 6 |
| BP | GO:0042255 | ribosome assembly | 9/799 | 64/18862 | 0.001430579 | 0.012892522 | 9 |
| BP | GO:0006301 | postreplication repair | 8/799 | 52/18862 | 0.001442182 | 0.012927955 | 8 |
| BP | GO:0048255 | mRNA stabilization | 8/799 | 52/18862 | 0.001442182 | 0.012927955 | 8 |
| BP | GO:0016575 | histone deacetylation | 10/799 | 77/18862 | 0.001479002 | 0.013187875 | 10 |
| BP | GO:0046785 | microtubule polymerization | 10/799 | 77/18862 | 0.001479002 | 0.013187875 | 10 |
| BP | GO:0002097 | tRNA wobble base modification | 5/799 | 21/18862 | 0.001555515 | 0.013724883 | 5 |
| BP | GO:0006293 | nucleotide-excision repair, preincision complex stabilization | 5/799 | 21/18862 | 0.001555515 | 0.013724883 | 5 |
| BP | GO:0006295 | nucleotide-excision repair, DNA incision, 3'-to lesion | 5/799 | 21/18862 | 0.001555515 | 0.013724883 | 5 |
| BP | GO:0031061 | negative regulation of histone methylation | 5/799 | 21/18862 | 0.001555515 | 0.013724883 | 5 |
| BP | GO:0034502 | protein localization to chromosome | 11/799 | 91/18862 | 0.001586375 | 0.013924272 | 11 |
| BP | GO:0048524 | positive regulation of viral process | 11/799 | 91/18862 | 0.001586375 | 0.013924272 | 11 |
| BP | GO:0090307 | mitotic spindle assembly | 9/799 | 65/18862 | 0.001599321 | 0.014001435 | 9 |
| BP | GO:0031453 | positive regulation of heterochromatin assembly | 4/799 | 13/18862 | 0.001681519 | 0.014644971 | 4 |
| BP | GO:0120263 | positive regulation of heterochromatin organization | 4/799 | 13/18862 | 0.001681519 | 0.014644971 | 4 |
| BP | GO:0030330 | DNA damage response, signal transduction by p53 class mediator | 12/799 | 106/18862 | 0.001757404 | 0.015266437 | 12 |
| BP | GO:0034724 | DNA replication-independent nucleosome organization | 8/799 | 54/18862 | 0.001851283 | 0.01595856 | 8 |
| BP | GO:0051784 | negative regulation of nuclear division | 8/799 | 54/18862 | 0.001851283 | 0.01595856 | 8 |
| BP | GO:0072698 | protein localization to microtubule cytoskeleton | 8/799 | 54/18862 | 0.001851283 | 0.01595856 | 8 |
| BP | GO:0042176 | regulation of protein catabolic process | 29/799 | 383/18862 | 0.001904297 | 0.016337773 | 29 |
| BP | GO:0006275 | regulation of DNA replication | 12/799 | 107/18862 | 0.001904968 | 0.016337773 | 12 |
| BP | GO:0000717 | nucleotide-excision repair, DNA duplex unwinding | 5/799 | 22/18862 | 0.001943576 | 0.016626586 | 5 |
| BP | GO:0006446 | regulation of translational initiation | 10/799 | 80/18862 | 0.001981651 | 0.016909385 | 10 |
| BP | GO:0046825 | regulation of protein export from nucleus | 6/799 | 32/18862 | 0.001998498 | 0.017010075 | 6 |
| BP | GO:0070507 | regulation of microtubule cytoskeleton organization | 14/799 | 137/18862 | 0.002040496 | 0.017323796 | 14 |
| BP | GO:0071156 | regulation of cell cycle arrest | 12/799 | 108/18862 | 0.002062538 | 0.017444425 | 12 |
| BP | GO:0032386 | regulation of intracellular transport | 27/799 | 350/18862 | 0.002065056 | 0.017444425 | 27 |
| BP | GO:0043967 | histone H4 acetylation | 9/799 | 68/18862 | 0.002203209 | 0.018564932 | 9 |
| BP | GO:0048608 | reproductive structure development | 30/799 | 405/18862 | 0.002239167 | 0.018754396 | 30 |
| BP | GO:0045799 | positive regulation of chromatin assembly or disassembly | 4/799 | 14/18862 | 0.002275772 | 0.018754396 | 4 |
| BP | GO:0051290 | protein heterotetramerization | 4/799 | 14/18862 | 0.002275772 | 0.018754396 | 4 |
| BP | GO:0072350 | tricarboxylic acid metabolic process | 4/799 | 14/18862 | 0.002275772 | 0.018754396 | 4 |
| BP | GO:0110156 | methylguanosine-cap decapping | 4/799 | 14/18862 | 0.002275772 | 0.018754396 | 4 |
| BP | GO:1901836 | regulation of transcription of nucleolar large rRNA by RNA polymerase I | 4/799 | 14/18862 | 0.002275772 | 0.018754396 | 4 |
| BP | GO:1902570 | protein localization to nucleolus | 4/799 | 14/18862 | 0.002275772 | 0.018754396 | 4 |
| BP | GO:1905168 | positive regulation of double-strand break repair via homologous recombination | 4/799 | 14/18862 | 0.002275772 | 0.018754396 | 4 |
| BP | GO:1990173 | protein localization to nucleoplasm | 4/799 | 14/18862 | 0.002275772 | 0.018754396 | 4 |
| BP | GO:0060271 | cilium assembly | 29/799 | 388/18862 | 0.002305563 | 0.01895356 | 29 |
| BP | GO:0006356 | regulation of transcription by RNA polymerase I | 6/799 | 33/18862 | 0.002356403 | 0.019277476 | 6 |
| BP | GO:0050685 | positive regulation of mRNA processing | 6/799 | 33/18862 | 0.002356403 | 0.019277476 | 6 |
| BP | GO:0000715 | nucleotide-excision repair, DNA damage recognition | 5/799 | 23/18862 | 0.002397884 | 0.019569325 | 5 |
| BP | GO:0009150 | purine ribonucleotide metabolic process | 30/799 | 408/18862 | 0.002499654 | 0.020301563 | 30 |
| BP | GO:0061458 | reproductive system development | 30/799 | 408/18862 | 0.002499654 | 0.020301563 | 30 |
| BP | GO:0045069 | regulation of viral genome replication | 10/799 | 83/18862 | 0.002613737 | 0.021177087 | 10 |
| BP | GO:0036297 | interstrand cross-link repair | 8/799 | 57/18862 | 0.002630686 | 0.021263304 | 8 |
| BP | GO:0006418 | tRNA aminoacylation for protein translation | 7/799 | 45/18862 | 0.002653004 | 0.021341336 | 7 |
| BP | GO:0031057 | negative regulation of histone modification | 7/799 | 45/18862 | 0.002653004 | 0.021341336 | 7 |
| BP | GO:0006891 | intra-Golgi vesicle-mediated transport | 6/799 | 34/18862 | 0.002760456 | 0.021995717 | 6 |
| BP | GO:0009112 | nucleobase metabolic process | 6/799 | 34/18862 | 0.002760456 | 0.021995717 | 6 |
| BP | GO:0009156 | ribonucleoside monophosphate biosynthetic process | 6/799 | 34/18862 | 0.002760456 | 0.021995717 | 6 |
| BP | GO:0051567 | histone H3-K9 methylation | 6/799 | 34/18862 | 0.002760456 | 0.021995717 | 6 |
| BP | GO:0060249 | anatomical structure homeostasis | 33/799 | 466/18862 | 0.002847323 | 0.022591349 | 33 |
| BP | GO:0010212 | response to ionizing radiation | 14/799 | 142/18862 | 0.002848613 | 0.022591349 | 14 |
| BP | GO:0043486 | histone exchange | 8/799 | 58/18862 | 0.002940692 | 0.023158128 | 8 |
| BP | GO:0044380 | protein localization to cytoskeleton | 8/799 | 58/18862 | 0.002940692 | 0.023158128 | 8 |
| BP | GO:0045815 | positive regulation of gene expression, epigenetic | 8/799 | 58/18862 | 0.002940692 | 0.023158128 | 8 |
| BP | GO:0000478 | endonucleolytic cleavage involved in rRNA processing | 4/799 | 15/18862 | 0.003000192 | 0.023462241 | 4 |
| BP | GO:0000479 | endonucleolytic cleavage of tricistronic rRNA transcript (SSU-rRNA, 5.8S rRNA, LSU-rRNA) | 4/799 | 15/18862 | 0.003000192 | 0.023462241 | 4 |
| BP | GO:0010452 | histone H3-K36 methylation | 4/799 | 15/18862 | 0.003000192 | 0.023462241 | 4 |
| BP | GO:2000045 | regulation of G1/S transition of mitotic cell cycle | 16/799 | 174/18862 | 0.003015563 | 0.023527856 | 16 |
| BP | GO:0051865 | protein autoubiquitination | 9/799 | 72/18862 | 0.003276171 | 0.025502127 | 9 |
| BP | GO:0031058 | positive regulation of histone modification | 10/799 | 86/18862 | 0.003397766 | 0.026362014 | 10 |
| BP | GO:0016073 | snRNA metabolic process | 7/799 | 47/18862 | 0.003417923 | 0.026362014 | 7 |
| BP | GO:0016574 | histone ubiquitination | 7/799 | 47/18862 | 0.003417923 | 0.026362014 | 7 |
| BP | GO:0031055 | chromatin remodeling at centromere | 7/799 | 47/18862 | 0.003417923 | 0.026362014 | 7 |
| BP | GO:0010639 | negative regulation of organelle organization | 26/799 | 346/18862 | 0.003521016 | 0.027095152 | 26 |
| BP | GO:0033119 | negative regulation of RNA splicing | 5/799 | 25/18862 | 0.003530141 | 0.027103492 | 5 |
| BP | GO:0019827 | stem cell population maintenance | 14/799 | 146/18862 | 0.003669722 | 0.028111123 | 14 |
| BP | GO:0009895 | negative regulation of catabolic process | 24/799 | 312/18862 | 0.003683998 | 0.028156489 | 24 |
| BP | GO:0031062 | positive regulation of histone methylation | 6/799 | 36/18862 | 0.003721313 | 0.028377341 | 6 |
| BP | GO:0006206 | pyrimidine nucleobase metabolic process | 4/799 | 16/18862 | 0.003867521 | 0.029293438 | 4 |
| BP | GO:0110154 | RNA decapping | 4/799 | 16/18862 | 0.003867521 | 0.029293438 | 4 |
| BP | GO:1902969 | mitotic DNA replication | 4/799 | 16/18862 | 0.003867521 | 0.029293438 | 4 |
| BP | GO:1902806 | regulation of cell cycle G1/S phase transition | 17/799 | 195/18862 | 0.003946558 | 0.029769057 | 17 |
| BP | GO:0045739 | positive regulation of DNA repair | 9/799 | 74/18862 | 0.003947979 | 0.029769057 | 9 |
| BP | GO:0098727 | maintenance of cell number | 14/799 | 148/18862 | 0.004147308 | 0.031202253 | 14 |
| BP | GO:0006163 | purine nucleotide metabolic process | 31/799 | 441/18862 | 0.004184571 | 0.031412485 | 31 |
| BP | GO:0043903 | regulation of biological process involved in symbiotic interaction | 17/799 | 197/18862 | 0.004375014 | 0.032769109 | 17 |
| BP | GO:2000756 | regulation of peptidyl-lysine acetylation | 8/799 | 62/18862 | 0.004474299 | 0.033438457 | 8 |
| BP | GO:0000018 | regulation of DNA recombination | 11/799 | 104/18862 | 0.004560999 | 0.03401099 | 11 |
| BP | GO:2000134 | negative regulation of G1/S transition of mitotic cell cycle | 12/799 | 119/18862 | 0.004608935 | 0.034292572 | 12 |
| BP | GO:0006368 | transcription elongation from RNA polymerase II promoter | 10/799 | 90/18862 | 0.004721633 | 0.034986537 | 10 |
| BP | GO:1901983 | regulation of protein acetylation | 9/799 | 76/18862 | 0.004722964 | 0.034986537 | 9 |
| BP | GO:0044782 | cilium organization | 29/799 | 409/18862 | 0.004876249 | 0.035829951 | 29 |
| BP | GO:0000963 | mitochondrial RNA processing | 4/799 | 17/18862 | 0.004889972 | 0.035829951 | 4 |
| BP | GO:0006390 | mitochondrial transcription | 4/799 | 17/18862 | 0.004889972 | 0.035829951 | 4 |
| BP | GO:0035518 | histone H2A monoubiquitination | 4/799 | 17/18862 | 0.004889972 | 0.035829951 | 4 |
| BP | GO:0051382 | kinetochore assembly | 4/799 | 17/18862 | 0.004889972 | 0.035829951 | 4 |
| BP | GO:1904029 | regulation of cyclin-dependent protein kinase activity | 11/799 | 105/18862 | 0.004905233 | 0.035863813 | 11 |
| BP | GO:0000469 | cleavage involved in rRNA processing | 5/799 | 27/18862 | 0.005002141 | 0.036335877 | 5 |
| BP | GO:0070199 | establishment of protein localization to chromosome | 5/799 | 27/18862 | 0.005002141 | 0.036335877 | 5 |
| BP | GO:1904357 | negative regulation of telomere maintenance via telomere lengthening | 5/799 | 27/18862 | 0.005002141 | 0.036335877 | 5 |
| BP | GO:0009314 | response to radiation | 31/799 | 447/18862 | 0.005083891 | 0.0368503 | 31 |
| BP | GO:0042769 | DNA damage response, detection of DNA damage | 6/799 | 39/18862 | 0.005599223 | 0.040411836 | 6 |
| BP | GO:1901998 | toxin transport | 6/799 | 39/18862 | 0.005599223 | 0.040411836 | 6 |
| BP | GO:2001022 | positive regulation of response to DNA damage stimulus | 11/799 | 107/18862 | 0.005655263 | 0.040729087 | 11 |
| BP | GO:0032388 | positive regulation of intracellular transport | 18/799 | 219/18862 | 0.005684976 | 0.04085578 | 18 |
| BP | GO:0000027 | ribosomal large subunit assembly | 5/799 | 28/18862 | 0.005880957 | 0.042084755 | 5 |
| BP | GO:0008053 | mitochondrial fusion | 5/799 | 28/18862 | 0.005880957 | 0.042084755 | 5 |
| BP | GO:0000387 | spliceosomal snRNP assembly | 7/799 | 52/18862 | 0.006060739 | 0.042776431 | 7 |
| BP | GO:0001522 | pseudouridine synthesis | 4/799 | 18/18862 | 0.006079143 | 0.042776431 | 4 |
| BP | GO:0002098 | tRNA wobble uridine modification | 4/799 | 18/18862 | 0.006079143 | 0.042776431 | 4 |
| BP | GO:0019054 | modulation by virus of host cellular process | 4/799 | 18/18862 | 0.006079143 | 0.042776431 | 4 |
| BP | GO:0031445 | regulation of heterochromatin assembly | 4/799 | 18/18862 | 0.006079143 | 0.042776431 | 4 |
| BP | GO:0046112 | nucleobase biosynthetic process | 4/799 | 18/18862 | 0.006079143 | 0.042776431 | 4 |
| BP | GO:0070200 | establishment of protein localization to telomere | 4/799 | 18/18862 | 0.006079143 | 0.042776431 | 4 |
| BP | GO:0120261 | regulation of heterochromatin organization | 4/799 | 18/18862 | 0.006079143 | 0.042776431 | 4 |
| BP | GO:0000154 | rRNA modification | 6/799 | 40/18862 | 0.006356351 | 0.044633852 | 6 |
| BP | GO:1902807 | negative regulation of cell cycle G1/S phase transition | 12/799 | 124/18862 | 0.006393241 | 0.044799554 | 12 |
| BP | GO:0006476 | protein deacetylation | 10/799 | 94/18862 | 0.006420566 | 0.04489769 | 10 |
| BP | GO:0006513 | protein monoubiquitination | 8/799 | 66/18862 | 0.006558634 | 0.045768214 | 8 |
| BP | GO:0000245 | spliceosomal complex assembly | 9/799 | 80/18862 | 0.00662341 | 0.04612475 | 9 |
| BP | GO:0000731 | DNA synthesis involved in DNA repair | 7/799 | 53/18862 | 0.00673397 | 0.046701697 | 7 |
| BP | GO:0006336 | DNA replication-independent nucleosome assembly | 7/799 | 53/18862 | 0.00673397 | 0.046701697 | 7 |
| BP | GO:0010390 | histone monoubiquitination | 5/799 | 29/18862 | 0.006863018 | 0.047498941 | 5 |
| BP | GO:0006188 | IMP biosynthetic process | 3/799 | 10/18862 | 0.007266879 | 0.049782944 | 3 |
| BP | GO:0006222 | UMP biosynthetic process | 3/799 | 10/18862 | 0.007266879 | 0.049782944 | 3 |
| BP | GO:0009174 | pyrimidine ribonucleoside monophosphate biosynthetic process | 3/799 | 10/18862 | 0.007266879 | 0.049782944 | 3 |
| BP | GO:0046487 | glyoxylate metabolic process | 3/799 | 10/18862 | 0.007266879 | 0.049782944 | 3 |
| BP | GO:0051573 | negative regulation of histone H3-K9 methylation | 3/799 | 10/18862 | 0.007266879 | 0.049782944 | 3 |

**Table S5b. CC of GO enrichment analysis.**

| ONTOLOGY | ID | Description | GeneRatio | BgRatio | pvalue | qvalue | Count |
| --- | --- | --- | --- | --- | --- | --- | --- |
| CC | GO:0005643 | nuclear pore | 26/808 | 85/19520 | 3.66E-16 | 1.43E-13 | 26 |
| CC | GO:0018995 | host cellular component | 21/808 | 60/19520 | 1.21E-14 | 1.58E-12 | 21 |
| CC | GO:0043657 | host cell | 21/808 | 60/19520 | 1.21E-14 | 1.58E-12 | 21 |
| CC | GO:0005635 | nuclear envelope | 56/808 | 462/19520 | 5.25E-13 | 5.14E-11 | 56 |
| CC | GO:0000228 | nuclear chromosome | 39/808 | 250/19520 | 8.29E-13 | 6.49E-11 | 39 |
| CC | GO:0016607 | nuclear speck | 49/808 | 411/19520 | 2.83E-11 | 1.81E-09 | 49 |
| CC | GO:0098687 | chromosomal region | 44/808 | 345/19520 | 3.24E-11 | 1.81E-09 | 44 |
| CC | GO:0061695 | transferase complex, transferring phosphorus-containing groups | 36/808 | 253/19520 | 9.43E-11 | 4.62E-09 | 36 |
| CC | GO:0009295 | nucleoid | 15/808 | 44/19520 | 1.18E-10 | 4.63E-09 | 15 |
| CC | GO:0042645 | mitochondrial nucleoid | 15/808 | 44/19520 | 1.18E-10 | 4.63E-09 | 15 |
| CC | GO:0005667 | transcription regulator complex | 47/808 | 409/19520 | 2.54E-10 | 9.05E-09 | 47 |
| CC | GO:0005681 | spliceosomal complex | 30/808 | 191/19520 | 3.08E-10 | 1.01E-08 | 30 |
| CC | GO:0005849 | mRNA cleavage factor complex | 10/808 | 22/19520 | 5.75E-09 | 1.73E-07 | 10 |
| CC | GO:0070603 | SWI/SNF superfamily-type complex | 17/808 | 74/19520 | 6.51E-09 | 1.75E-07 | 17 |
| CC | GO:0031965 | nuclear membrane | 36/808 | 295/19520 | 6.69E-09 | 1.75E-07 | 36 |
| CC | GO:0000428 | DNA-directed RNA polymerase complex | 20/808 | 104/19520 | 8.27E-09 | 2.02E-07 | 20 |
| CC | GO:0005847 | mRNA cleavage and polyadenylation specificity factor complex | 9/808 | 18/19520 | 1.19E-08 | 2.73E-07 | 9 |
| CC | GO:0030880 | RNA polymerase complex | 20/808 | 108/19520 | 1.63E-08 | 3.54E-07 | 20 |
| CC | GO:0031080 | nuclear pore outer ring | 7/808 | 10/19520 | 2.18E-08 | 4.50E-07 | 7 |
| CC | GO:0055029 | nuclear DNA-directed RNA polymerase complex | 19/808 | 103/19520 | 3.95E-08 | 7.74E-07 | 19 |
| CC | GO:0000775 | chromosome, centromeric region | 27/808 | 196/19520 | 4.20E-08 | 7.84E-07 | 27 |
| CC | GO:0034708 | methyltransferase complex | 18/808 | 96/19520 | 6.91E-08 | 1.19E-06 | 18 |
| CC | GO:0005819 | spindle | 40/808 | 381/19520 | 6.98E-08 | 1.19E-06 | 40 |
| CC | GO:0071013 | catalytic step 2 spliceosome | 17/808 | 87/19520 | 8.47E-08 | 1.38E-06 | 17 |
| CC | GO:0090575 | RNA polymerase II transcription regulator complex | 24/808 | 170/19520 | 1.48E-07 | 2.31E-06 | 24 |
| CC | GO:1904949 | ATPase complex | 18/808 | 103/19520 | 2.11E-07 | 3.18E-06 | 18 |
| CC | GO:0034399 | nuclear periphery | 20/808 | 128/19520 | 3.04E-07 | 4.41E-06 | 20 |
| CC | GO:0035770 | ribonucleoprotein granule | 29/808 | 244/19520 | 3.45E-07 | 4.82E-06 | 29 |
| CC | GO:0035097 | histone methyltransferase complex | 14/808 | 68/19520 | 5.97E-07 | 7.88E-06 | 14 |
| CC | GO:0030684 | preribosome | 15/808 | 78/19520 | 6.03E-07 | 7.88E-06 | 15 |
| CC | GO:0005759 | mitochondrial matrix | 43/808 | 476/19520 | 1.46E-06 | 1.84E-05 | 43 |
| CC | GO:0005657 | replication fork | 13/808 | 65/19520 | 2.13E-06 | 2.60E-05 | 13 |
| CC | GO:0000776 | kinetochore | 19/808 | 137/19520 | 3.77E-06 | 4.45E-05 | 19 |
| CC | GO:0000793 | condensed chromosome | 25/808 | 217/19520 | 3.86E-06 | 4.45E-05 | 25 |
| CC | GO:0000123 | histone acetyltransferase complex | 14/808 | 84/19520 | 8.32E-06 | 9.31E-05 | 14 |
| CC | GO:0071011 | precatalytic spliceosome | 11/808 | 53/19520 | 8.86E-06 | 9.63E-05 | 11 |
| CC | GO:0044665 | MLL1/2 complex | 8/808 | 28/19520 | 1.24E-05 | 0.000127477 | 8 |
| CC | GO:0071339 | MLL1 complex | 8/808 | 28/19520 | 1.24E-05 | 0.000127477 | 8 |
| CC | GO:0036464 | cytoplasmic ribonucleoprotein granule | 25/808 | 234/19520 | 1.46E-05 | 0.000146579 | 25 |
| CC | GO:0031011 | Ino80 complex | 6/808 | 15/19520 | 1.79E-05 | 0.000171207 | 6 |
| CC | GO:0033202 | DNA helicase complex | 6/808 | 15/19520 | 1.79E-05 | 0.000171207 | 6 |
| CC | GO:0016591 | RNA polymerase II, holoenzyme | 13/808 | 79/19520 | 2.00E-05 | 0.000186829 | 13 |
| CC | GO:0072686 | mitotic spindle | 19/808 | 157/19520 | 2.75E-05 | 0.00025068 | 19 |
| CC | GO:0031248 | protein acetyltransferase complex | 14/808 | 94/19520 | 3.12E-05 | 0.000271179 | 14 |
| CC | GO:1902493 | acetyltransferase complex | 14/808 | 94/19520 | 3.12E-05 | 0.000271179 | 14 |
| CC | GO:0071005 | U2-type precatalytic spliceosome | 10/808 | 50/19520 | 3.19E-05 | 0.000271277 | 10 |
| CC | GO:0097346 | INO80-type complex | 7/808 | 25/19520 | 5.07E-05 | 0.000422395 | 7 |
| CC | GO:0043596 | nuclear replication fork | 8/808 | 35/19520 | 7.24E-05 | 0.000589038 | 8 |
| CC | GO:0071564 | npBAF complex | 5/808 | 12/19520 | 7.46E-05 | 0.000589038 | 5 |
| CC | GO:0005814 | centriole | 17/808 | 141/19520 | 7.52E-05 | 0.000589038 | 17 |
| CC | GO:0000779 | condensed chromosome, centromeric region | 15/808 | 117/19520 | 9.76E-05 | 0.000749038 | 15 |
| CC | GO:1902562 | H4 histone acetyltransferase complex | 9/808 | 47/19520 | 0.000112909 | 0.000842685 | 9 |
| CC | GO:0005684 | U2-type spliceosomal complex | 13/808 | 93/19520 | 0.000115726 | 0.000842685 | 13 |
| CC | GO:0042575 | DNA polymerase complex | 6/808 | 20/19520 | 0.000116209 | 0.000842685 | 6 |
| CC | GO:0030686 | 90S preribosome | 7/808 | 29/19520 | 0.000142448 | 0.001014176 | 7 |
| CC | GO:0000930 | gamma-tubulin complex | 6/808 | 21/19520 | 0.000157022 | 0.00109797 | 6 |
| CC | GO:0016363 | nuclear matrix | 14/808 | 109/19520 | 0.000161771 | 0.001111334 | 14 |
| CC | GO:0000922 | spindle pole | 18/808 | 166/19520 | 0.000184929 | 0.001248519 | 18 |
| CC | GO:0000152 | nuclear ubiquitin ligase complex | 8/808 | 43/19520 | 0.000333088 | 0.002210683 | 8 |
| CC | GO:0030894 | replisome | 6/808 | 24/19520 | 0.000350212 | 0.002268545 | 6 |
| CC | GO:0030014 | CCR4-NOT complex | 5/808 | 16/19520 | 0.000358043 | 0.002268545 | 5 |
| CC | GO:0000932 | P-body | 12/808 | 91/19520 | 0.00036498 | 0.002268545 | 12 |
| CC | GO:0090734 | site of DNA damage | 12/808 | 91/19520 | 0.00036498 | 0.002268545 | 12 |
| CC | GO:0000781 | chromosome, telomeric region | 17/808 | 161/19520 | 0.000378381 | 0.002291502 | 17 |
| CC | GO:0005874 | microtubule | 33/808 | 423/19520 | 0.000388849 | 0.002291502 | 33 |
| CC | GO:0031304 | intrinsic component of mitochondrial inner membrane | 9/808 | 55/19520 | 0.000392081 | 0.002291502 | 9 |
| CC | GO:0031305 | integral component of mitochondrial inner membrane | 9/808 | 55/19520 | 0.000392081 | 0.002291502 | 9 |
| CC | GO:0005669 | transcription factor TFIID complex | 7/808 | 34/19520 | 0.000410003 | 0.002361009 | 7 |
| CC | GO:0000777 | condensed chromosome kinetochore | 13/808 | 106/19520 | 0.000433532 | 0.002460318 | 13 |
| CC | GO:0071162 | CMG complex | 4/808 | 10/19520 | 0.000500993 | 0.002763074 | 4 |
| CC | GO:0097550 | transcription preinitiation complex | 4/808 | 10/19520 | 0.000500993 | 0.002763074 | 4 |
| CC | GO:0035861 | site of double-strand break | 10/808 | 69/19520 | 0.000519495 | 0.002825323 | 10 |
| CC | GO:0032592 | integral component of mitochondrial membrane | 11/808 | 85/19520 | 0.000745213 | 0.003997394 | 11 |
| CC | GO:0005832 | chaperonin-containing T-complex | 4/808 | 11/19520 | 0.000761546 | 0.004029805 | 4 |
| CC | GO:0010494 | cytoplasmic stress granule | 10/808 | 73/19520 | 0.000818307 | 0.004241361 | 10 |
| CC | GO:0098573 | intrinsic component of mitochondrial membrane | 11/808 | 86/19520 | 0.000823189 | 0.004241361 | 11 |
| CC | GO:0016514 | SWI/SNF complex | 5/808 | 19/19520 | 0.000859527 | 0.004371075 | 5 |
| CC | GO:0016234 | inclusion body | 10/808 | 74/19520 | 0.000911736 | 0.004519199 | 10 |
| CC | GO:0017053 | transcription repressor complex | 10/808 | 74/19520 | 0.000911736 | 0.004519199 | 10 |
| CC | GO:0032040 | small-subunit processome | 7/808 | 39/19520 | 0.000979501 | 0.0047944 | 7 |
| CC | GO:0000109 | nucleotide-excision repair complex | 4/808 | 12/19520 | 0.001105043 | 0.005151328 | 4 |
| CC | GO:0031261 | DNA replication preinitiation complex | 4/808 | 12/19520 | 0.001105043 | 0.005151328 | 4 |
| CC | GO:0031464 | Cul4A-RING E3 ubiquitin ligase complex | 4/808 | 12/19520 | 0.001105043 | 0.005151328 | 4 |
| CC | GO:0042405 | nuclear inclusion body | 4/808 | 12/19520 | 0.001105043 | 0.005151328 | 4 |
| CC | GO:1990391 | DNA repair complex | 7/808 | 40/19520 | 0.001145462 | 0.005276929 | 7 |
| CC | GO:0015030 | Cajal body | 10/808 | 78/19520 | 0.001377093 | 0.006270239 | 10 |
| CC | GO:0043601 | nuclear replisome | 5/808 | 22/19520 | 0.00175599 | 0.007903551 | 5 |
| CC | GO:0001650 | fibrillar center | 14/808 | 139/19520 | 0.001893888 | 0.008427348 | 14 |
| CC | GO:0030687 | preribosome, large subunit precursor | 5/808 | 23/19520 | 0.002168176 | 0.00949606 | 5 |
| CC | GO:0000151 | ubiquitin ligase complex | 23/808 | 289/19520 | 0.002182562 | 0.00949606 | 23 |
| CC | GO:0005671 | Ada2/Gcn5/Ada3 transcription activator complex | 4/808 | 15/19520 | 0.002759376 | 0.011873774 | 4 |
| CC | GO:0000791 | euchromatin | 6/808 | 36/19520 | 0.003322209 | 0.014140294 | 6 |
| CC | GO:0070822 | Sin3-type complex | 4/808 | 16/19520 | 0.00355982 | 0.014988716 | 4 |
| CC | GO:0005743 | mitochondrial inner membrane | 33/808 | 493/19520 | 0.004751811 | 0.019794777 | 33 |
| CC | GO:0032039 | integrator complex | 5/808 | 28/19520 | 0.005338638 | 0.021776024 | 5 |
| CC | GO:0101031 | chaperone complex | 5/808 | 28/19520 | 0.005338638 | 0.021776024 | 5 |
| CC | GO:0016592 | mediator complex | 6/808 | 40/19520 | 0.005692907 | 0.022981674 | 6 |
| CC | GO:0032993 | protein-DNA complex | 17/808 | 208/19520 | 0.005995678 | 0.023956952 | 17 |
| CC | GO:0030496 | midbody | 16/808 | 193/19520 | 0.006601292 | 0.026110374 | 16 |
| CC | GO:0120114 | Sm-like protein family complex | 11/808 | 113/19520 | 0.007177281 | 0.028104723 | 11 |
| CC | GO:0031461 | cullin-RING ubiquitin ligase complex | 14/808 | 163/19520 | 0.007904525 | 0.030645996 | 14 |
| CC | GO:0008023 | transcription elongation factor complex | 7/808 | 56/19520 | 0.008057908 | 0.030934383 | 7 |
| CC | GO:0000242 | pericentriolar material | 4/808 | 20/19520 | 0.008309736 | 0.031382596 | 4 |
| CC | GO:1902555 | endoribonuclease complex | 5/808 | 31/19520 | 0.008334948 | 0.031382596 | 5 |
| CC | GO:0030532 | small nuclear ribonucleoprotein complex | 10/808 | 101/19520 | 0.009019703 | 0.033565377 | 10 |
| CC | GO:0031010 | ISWI-type complex | 3/808 | 11/19520 | 0.009086111 | 0.033565377 | 3 |
| CC | GO:1990752 | microtubule end | 5/808 | 32/19520 | 0.009549125 | 0.034946134 | 5 |
| CC | GO:1902911 | protein kinase complex | 10/808 | 104/19520 | 0.010980684 | 0.039813008 | 10 |
| CC | GO:1902554 | serine/threonine protein kinase complex | 9/808 | 89/19520 | 0.01131522 | 0.040367023 | 9 |
| CC | GO:0042555 | MCM complex | 3/808 | 12/19520 | 0.011746468 | 0.040367023 | 3 |
| CC | GO:0044615 | nuclear pore nuclear basket | 3/808 | 12/19520 | 0.011746468 | 0.040367023 | 3 |
| CC | GO:0005680 | anaphase-promoting complex | 4/808 | 22/19520 | 0.011752012 | 0.040367023 | 4 |
| CC | GO:0005697 | telomerase holoenzyme complex | 4/808 | 22/19520 | 0.011752012 | 0.040367023 | 4 |
| CC | GO:0035371 | microtubule plus-end | 4/808 | 22/19520 | 0.011752012 | 0.040367023 | 4 |
| CC | GO:0097431 | mitotic spindle pole | 5/808 | 34/19520 | 0.012331159 | 0.041988018 | 5 |
| CC | GO:0031519 | PcG protein complex | 6/808 | 47/19520 | 0.012486155 | 0.042149271 | 6 |
| CC | GO:0097525 | spliceosomal snRNP complex | 9/808 | 91/19520 | 0.012978639 | 0.04343728 | 9 |
| CC | GO:0098799 | outer mitochondrial membrane protein complex | 4/808 | 23/19520 | 0.013769662 | 0.045694151 | 4 |
| CC | GO:0080008 | Cul4-RING E3 ubiquitin ligase complex | 5/808 | 35/19520 | 0.013908488 | 0.045766983 | 5 |
| CC | GO:0000118 | histone deacetylase complex | 8/808 | 77/19520 | 0.014152526 | 0.046181928 | 8 |
| CC | GO:0000974 | Prp19 complex | 3/808 | 13/19520 | 0.014807159 | 0.04675945 | 3 |
| CC | GO:0005662 | DNA replication factor A complex | 3/808 | 13/19520 | 0.014807159 | 0.04675945 | 3 |
| CC | GO:0016580 | Sin3 complex | 3/808 | 13/19520 | 0.014807159 | 0.04675945 | 3 |
| CC | GO:0044666 | MLL3/4 complex | 3/808 | 13/19520 | 0.014807159 | 0.04675945 | 3 |
| CC | GO:0051233 | spindle midzone | 5/808 | 36/19520 | 0.015615952 | 0.048919024 | 5 |
| CC | GO:0000178 | exosome (RNase complex) | 4/808 | 24/19520 | 0.015994277 | 0.049706525 | 4 |

**Table S5c. MF of GO enrichment analysis.**

| ONTOLOGY | ID | Description | GeneRatio | BgRatio | pvalue | qvalue | Count |
| --- | --- | --- | --- | --- | --- | --- | --- |
| MF | GO:0140098 | catalytic activity, acting on RNA | 59/804 | 386/18337 | 3.59E-17 | 1.96E-14 | 59 |
| MF | GO:0017056 | structural constituent of nuclear pore | 15/804 | 28/18337 | 8.23E-14 | 2.25E-11 | 15 |
| MF | GO:0004386 | helicase activity | 32/804 | 157/18337 | 2.44E-13 | 4.45E-11 | 32 |
| MF | GO:0016887 | ATPase activity | 58/804 | 478/18337 | 1.97E-12 | 2.56E-10 | 58 |
| MF | GO:0003712 | transcription coregulator activity | 58/804 | 480/18337 | 2.35E-12 | 2.56E-10 | 58 |
| MF | GO:0003713 | transcription coactivator activity | 36/804 | 258/18337 | 7.72E-10 | 7.03E-08 | 36 |
| MF | GO:0042393 | histone binding | 34/804 | 241/18337 | 1.71E-09 | 1.33E-07 | 34 |
| MF | GO:0140097 | catalytic activity, acting on DNA | 30/804 | 204/18337 | 5.81E-09 | 3.97E-07 | 30 |
| MF | GO:0003724 | RNA helicase activity | 16/804 | 77/18337 | 1.79E-07 | 1.09E-05 | 16 |
| MF | GO:0030374 | nuclear receptor coactivator activity | 13/804 | 54/18337 | 4.20E-07 | 2.30E-05 | 13 |
| MF | GO:0140142 | nucleocytoplasmic carrier activity | 10/804 | 31/18337 | 4.76E-07 | 2.36E-05 | 10 |
| MF | GO:0140297 | DNA-binding transcription factor binding | 39/804 | 376/18337 | 5.92E-07 | 2.69E-05 | 39 |
| MF | GO:0003697 | single-stranded DNA binding | 19/804 | 116/18337 | 6.68E-07 | 2.81E-05 | 19 |
| MF | GO:0061629 | RNA polymerase II-specific DNA-binding transcription factor binding | 31/804 | 271/18337 | 1.08E-06 | 4.23E-05 | 31 |
| MF | GO:0008139 | nuclear localization sequence binding | 9/804 | 27/18337 | 1.32E-06 | 4.79E-05 | 9 |
| MF | GO:0140030 | modification-dependent protein binding | 22/804 | 157/18337 | 1.42E-06 | 4.81E-05 | 22 |
| MF | GO:0043021 | ribonucleoprotein complex binding | 20/804 | 134/18337 | 1.56E-06 | 4.81E-05 | 20 |
| MF | GO:0008757 | S-adenosylmethionine-dependent methyltransferase activity | 22/804 | 158/18337 | 1.59E-06 | 4.81E-05 | 22 |
| MF | GO:0016741 | transferase activity, transferring one-carbon groups | 27/804 | 226/18337 | 2.32E-06 | 6.54E-05 | 27 |
| MF | GO:0004527 | exonuclease activity | 15/804 | 82/18337 | 2.39E-06 | 6.54E-05 | 15 |
| MF | GO:0008168 | methyltransferase activity | 26/804 | 215/18337 | 2.83E-06 | 7.36E-05 | 26 |
| MF | GO:0008408 | 3'-5' exonuclease activity | 12/804 | 55/18337 | 3.58E-06 | 8.90E-05 | 12 |
| MF | GO:0016796 | exonuclease activity, active with either ribo- or deoxyribonucleic acids and producing 5'-phosphomonoesters | 12/804 | 57/18337 | 5.33E-06 | 0.000126592 | 12 |
| MF | GO:0140101 | catalytic activity, acting on a tRNA | 18/804 | 123/18337 | 6.85E-06 | 0.000155825 | 18 |
| MF | GO:0051539 | 4 iron, 4 sulfur cluster binding | 10/804 | 42/18337 | 1.02E-05 | 0.0002226 | 10 |
| MF | GO:0004518 | nuclease activity | 24/804 | 206/18337 | 1.28E-05 | 0.000268852 | 24 |
| MF | GO:0000175 | 3'-5'-exoribonuclease activity | 9/804 | 35/18337 | 1.45E-05 | 0.000292526 | 9 |
| MF | GO:0003678 | DNA helicase activity | 13/804 | 74/18337 | 1.77E-05 | 0.000344375 | 13 |
| MF | GO:0008170 | N-methyltransferase activity | 15/804 | 98/18337 | 2.29E-05 | 0.000428718 | 15 |
| MF | GO:0016896 | exoribonuclease activity, producing 5'-phosphomonoesters | 9/804 | 37/18337 | 2.35E-05 | 0.000428718 | 9 |
| MF | GO:0046966 | thyroid hormone receptor binding | 8/804 | 29/18337 | 2.49E-05 | 0.0004389 | 8 |
| MF | GO:0003727 | single-stranded RNA binding | 14/804 | 88/18337 | 2.73E-05 | 0.000465851 | 14 |
| MF | GO:0003899 | DNA-directed 5'-3' RNA polymerase activity | 9/804 | 39/18337 | 3.71E-05 | 0.000595601 | 9 |
| MF | GO:0004532 | exoribonuclease activity | 9/804 | 39/18337 | 3.71E-05 | 0.000595601 | 9 |
| MF | GO:0035257 | nuclear hormone receptor binding | 18/804 | 140/18337 | 4.14E-05 | 0.000645683 | 18 |
| MF | GO:0031491 | nucleosome binding | 12/804 | 70/18337 | 4.75E-05 | 0.000721228 | 12 |
| MF | GO:0043138 | 3'-5' DNA helicase activity | 6/804 | 17/18337 | 5.69E-05 | 0.000840767 | 6 |
| MF | GO:0005049 | nuclear export signal receptor activity | 5/804 | 11/18337 | 5.93E-05 | 0.000852347 | 5 |
| MF | GO:0035064 | methylated histone binding | 12/804 | 72/18337 | 6.33E-05 | 0.00086506 | 12 |
| MF | GO:0140034 | methylation-dependent protein binding | 12/804 | 72/18337 | 6.33E-05 | 0.00086506 | 12 |
| MF | GO:0018024 | histone-lysine N-methyltransferase activity | 9/804 | 43/18337 | 8.43E-05 | 0.001070883 | 9 |
| MF | GO:0034062 | 5'-3' RNA polymerase activity | 9/804 | 43/18337 | 8.43E-05 | 0.001070883 | 9 |
| MF | GO:0097747 | RNA polymerase activity | 9/804 | 43/18337 | 8.43E-05 | 0.001070883 | 9 |
| MF | GO:0042054 | histone methyltransferase activity | 10/804 | 53/18337 | 8.72E-05 | 0.001083245 | 10 |
| MF | GO:0003684 | damaged DNA binding | 11/804 | 66/18337 | 0.000127095 | 0.001542973 | 11 |
| MF | GO:0140104 | molecular carrier activity | 11/804 | 67/18337 | 0.000146124 | 0.001730569 | 11 |
| MF | GO:0003714 | transcription corepressor activity | 20/804 | 182/18337 | 0.000148882 | 0.001730569 | 20 |
| MF | GO:0004535 | poly(A)-specific ribonuclease activity | 5/804 | 13/18337 | 0.000153455 | 0.001746565 | 5 |
| MF | GO:0016251 | RNA polymerase II general transcription initiation factor activity | 8/804 | 38/18337 | 0.000199995 | 0.002229799 | 8 |
| MF | GO:0016279 | protein-lysine N-methyltransferase activity | 10/804 | 60/18337 | 0.000255779 | 0.002794723 | 10 |
| MF | GO:0005048 | signal sequence binding | 9/804 | 50/18337 | 0.000284611 | 0.002984832 | 9 |
| MF | GO:0001046 | core promoter sequence-specific DNA binding | 8/804 | 40/18337 | 0.000291054 | 0.002984832 | 8 |
| MF | GO:0016278 | lysine N-methyltransferase activity | 10/804 | 61/18337 | 0.000294092 | 0.002984832 | 10 |
| MF | GO:0016874 | ligase activity | 18/804 | 163/18337 | 0.000295033 | 0.002984832 | 18 |
| MF | GO:0008276 | protein methyltransferase activity | 12/804 | 85/18337 | 0.000322318 | 0.003201589 | 12 |
| MF | GO:0017069 | snRNA binding | 9/804 | 52/18337 | 0.000386484 | 0.003743785 | 9 |
| MF | GO:0030515 | snoRNA binding | 7/804 | 32/18337 | 0.000390609 | 0.003743785 | 7 |
| MF | GO:0032182 | ubiquitin-like protein binding | 13/804 | 101/18337 | 0.000464133 | 0.004371783 | 13 |
| MF | GO:0002039 | p53 binding | 10/804 | 66/18337 | 0.000564977 | 0.005231459 | 10 |
| MF | GO:0051427 | hormone receptor binding | 18/804 | 173/18337 | 0.000607516 | 0.005531589 | 18 |
| MF | GO:0000400 | four-way junction DNA binding | 5/804 | 17/18337 | 0.000637305 | 0.005536525 | 5 |
| MF | GO:0051536 | iron-sulfur cluster binding | 10/804 | 67/18337 | 0.000638461 | 0.005536525 | 10 |
| MF | GO:0051540 | metal cluster binding | 10/804 | 67/18337 | 0.000638461 | 0.005536525 | 10 |
| MF | GO:0140223 | general transcription initiation factor activity | 8/804 | 45/18337 | 0.000672369 | 0.005739467 | 8 |
| MF | GO:0000217 | DNA secondary structure binding | 7/804 | 36/18337 | 0.000831751 | 0.00699075 | 7 |
| MF | GO:0000979 | RNA polymerase II core promoter sequence-specific DNA binding | 5/804 | 18/18337 | 0.000850786 | 0.007042392 | 5 |
| MF | GO:1990841 | promoter-specific chromatin binding | 9/804 | 58/18337 | 0.000885066 | 0.007216797 | 9 |
| MF | GO:0017116 | single-stranded DNA helicase activity | 5/804 | 19/18337 | 0.00111329 | 0.008944237 | 5 |
| MF | GO:0061608 | nuclear import signal receptor activity | 5/804 | 20/18337 | 0.001431292 | 0.011170538 | 5 |
| MF | GO:0070182 | DNA polymerase binding | 5/804 | 20/18337 | 0.001431292 | 0.011170538 | 5 |
| MF | GO:0004540 | ribonuclease activity | 13/804 | 114/18337 | 0.001464748 | 0.01127063 | 13 |
| MF | GO:0070577 | lysine-acetylated histone binding | 5/804 | 21/18337 | 0.001811456 | 0.013316643 | 5 |
| MF | GO:0140033 | acetylation-dependent protein binding | 5/804 | 21/18337 | 0.001811456 | 0.013316643 | 5 |
| MF | GO:0016779 | nucleotidyltransferase activity | 14/804 | 131/18337 | 0.001834171 | 0.013316643 | 14 |
| MF | GO:0004812 | aminoacyl-tRNA ligase activity | 7/804 | 41/18337 | 0.001852527 | 0.013316643 | 7 |
| MF | GO:0016875 | ligase activity, forming carbon-oxygen bonds | 7/804 | 41/18337 | 0.001852527 | 0.013316643 | 7 |
| MF | GO:0070491 | repressing transcription factor binding | 9/804 | 71/18337 | 0.003735964 | 0.026506703 | 9 |
| MF | GO:0008234 | cysteine-type peptidase activity | 16/804 | 174/18337 | 0.004203242 | 0.029439709 | 16 |
| MF | GO:0070063 | RNA polymerase binding | 9/804 | 73/18337 | 0.004507609 | 0.031171874 | 9 |
| MF | GO:0017025 | TBP-class protein binding | 5/804 | 26/18337 | 0.004885021 | 0.033359548 | 5 |
| MF | GO:0004197 | cysteine-type endopeptidase activity | 12/804 | 117/18337 | 0.005266175 | 0.035518452 | 12 |
| MF | GO:0031490 | chromatin DNA binding | 11/804 | 103/18337 | 0.005464459 | 0.036406343 | 11 |
| MF | GO:0051082 | unfolded protein binding | 12/804 | 120/18337 | 0.006435478 | 0.041940892 | 12 |
| MF | GO:0008094 | DNA-dependent ATPase activity | 7/804 | 51/18337 | 0.00653912 | 0.041940892 | 7 |
| MF | GO:0016830 | carbon-carbon lyase activity | 7/804 | 51/18337 | 0.00653912 | 0.041940892 | 7 |
| MF | GO:0043539 | protein serine/threonine kinase activator activity | 6/804 | 39/18337 | 0.006602256 | 0.041940892 | 6 |
| MF | GO:0031492 | nucleosomal DNA binding | 6/804 | 40/18337 | 0.007485777 | 0.047006877 | 6 |
| MF | GO:0034511 | U3 snoRNA binding | 3/804 | 10/18337 | 0.007995108 | 0.049077011 | 3 |
| MF | GO:0046974 | histone methyltransferase activity (H3-K9 specific) | 3/804 | 10/18337 | 0.007995108 | 0.049077011 | 3 |

# Appendix 6

## **KEGG enrichment analysis**

**Table 6. KEGG enrichment analysis.**

| ID | Description | GeneRatio | BgRatio | pvalue | qvalue | Count |
| --- | --- | --- | --- | --- | --- | --- |
| hsa03013 | Nucleocytoplasmic transport | 35/364 | 108/8163 | 1.59E-21 | 3.91E-19 | 35 |
| hsa03040 | Spliceosome | 25/364 | 147/8163 | 5.77E-09 | 5.47E-07 | 25 |
| hsa03015 | mRNA surveillance pathway | 20/364 | 97/8163 | 6.69E-09 | 5.47E-07 | 20 |
| hsa03030 | DNA replication | 11/364 | 36/8163 | 2.62E-07 | 1.61E-05 | 11 |
| hsa03420 | Nucleotide excision repair | 10/364 | 47/8163 | 3.24E-05 | 0.001590363 | 10 |
| hsa03410 | Base excision repair | 8/364 | 33/8163 | 7.53E-05 | 0.00307886 | 8 |
| hsa03022 | Basal transcription factors | 9/364 | 45/8163 | 0.000134334 | 0.004706741 | 9 |
| hsa03018 | RNA degradation | 12/364 | 79/8163 | 0.00017672 | 0.005417867 | 12 |
| hsa03008 | Ribosome biogenesis in eukaryotes | 14/364 | 109/8163 | 0.000323641 | 0.008819681 | 14 |
| hsa03430 | Mismatch repair | 6/364 | 23/8163 | 0.000399046 | 0.009787121 | 6 |
| hsa03020 | RNA polymerase | 7/364 | 34/8163 | 0.000626496 | 0.013968757 | 7 |
| hsa04110 | Cell cycle | 14/364 | 126/8163 | 0.001401139 | 0.028637323 | 14 |
| hsa05014 | Amyotrophic lateral sclerosis | 29/364 | 364/8163 | 0.001649777 | 0.031125339 | 29 |
| hsa03440 | Homologous recombination | 7/364 | 41/8163 | 0.002001904 | 0.035070945 | 7 |
| hsa00970 | Aminoacyl-tRNA biosynthesis | 9/364 | 66/8163 | 0.002459401 | 0.040213356 | 9 |
